# Antibody–drug conjugates in cancer therapy: mechanisms and clinical studies

**DOI:** 10.1002/mco2.671

**Published:** 2024-07-28

**Authors:** Jun He, Xianghua Zeng, Chunmei Wang, Enwen Wang, Yongsheng Li

**Affiliations:** ^1^ Department of General Surgery Jiande Branch of the Second Affiliated Hospital, School of Medicine, Zhejiang University Jiande Zhejiang China; ^2^ Department of Medical Oncology Chongqing University Cancer Hospital Chongqing China

**Keywords:** antibody–drug conjugates, cancer, clinical trials, cluster of differentiation, human epidermal growth factor receptor 2, mechanism, resistance, target antigen

## Abstract

Antibody–drug conjugates (ADCs) consist of monoclonal antibodies that target tumor cells and cytotoxic drugs linked through linkers. By leveraging antibodies’ targeting properties, ADCs deliver cytotoxic drugs into tumor cells via endocytosis after identifying the tumor antigen. This precise method aims to kill tumor cells selectively while minimizing harm to normal cells, offering safe and effective therapeutic benefits. Recent years have seen significant progress in antitumor treatment with ADC development, providing patients with new and potent treatment options. With over 300 ADCs explored for various tumor indications and some already approved for clinical use, challenges such as resistance due to factors like antigen expression, ADC processing, and payload have emerged. This review aims to outline the history of ADC development, their structure, mechanism of action, recent composition advancements, target selection, completed and ongoing clinical trials, resistance mechanisms, and intervention strategies. Additionally, it will delve into the potential of ADCs with novel markers, linkers, payloads, and innovative action mechanisms to enhance cancer treatment options. The evolution of ADCs has also led to the emergence of combination therapy as a new therapeutic approach to improve drug efficacy.

## Graphical abstract

The antibodies of ADCs bind to antigens on the cell surface, forming ADC–antigen complexes. These complexes are subsequently internalized into lysosomes via receptor‐mediated endocytosis. Once inside the lysosomes, the ADCs can be degraded by enzymes or the acidic environment, releasing cytotoxic payloads that can disrupt DNA or inhibit microtubule polymerization.

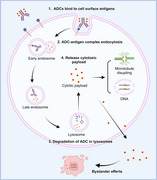

## 1 INTRODUCTION

Since the proposal of the “magic bullet” concept by German scientist Paul Ehrlich in 1913,^1^ the development of antibody‐conjugated drugs (ADCs) has experienced both successes and setbacks. ADCs are drug molecules that combine highly targeted antibody molecules with highly cytotoxic small molecules.^2^

**FIGURE 1:**
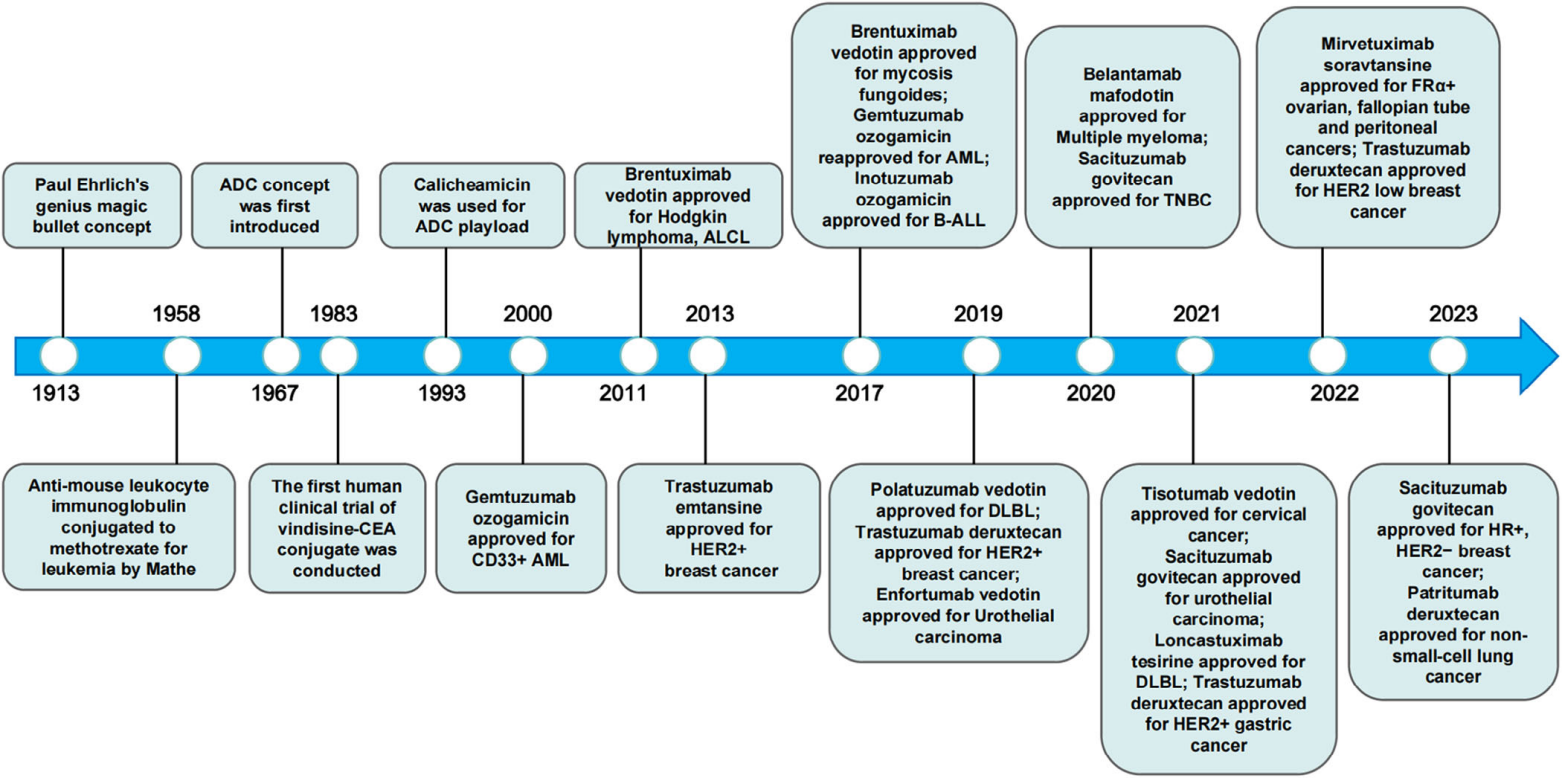
Timeline of landmark event in antibody–drug conjugates development over the past 100 years since the “magic bullet” proposed in 1910. ADC, antibody–drug conjugate; ALCL, anaplastic large‐cell lymphoma; AML, acute myeloid lymphoma; B‐ALL, B cell acute lymphoblastic leukemia; DLBL: diffuse large B cell lymphoma; HER2, human epidermal growth factor receptor‐2; FRα, folate receptor‐α; HR, hormone receptor; TNBC, triple‐negative breast cancer.

However, the concept of ADC was initially proposed in 1967,^3^ and it was not until 2000 that the first ADC drug, gemtuzumab ozogamicin (GO), was approved by the United States Food and Drug Administration (US FDA) for treating acute myeloid leukemia (AML).^4^ However, due to subsequent clinical findings indicating modest benefits but increased mortality, particularly from hepatic veno‐occlusive disease, GO was later withdrawn.^5^ Subsequently, there was limited progress in ADC drug development during the following decade. The second ADC drug was launched in 2010 and approved by the US FDA in 2011 for the treatment of Hodgkin lymphoma and systemic anaplastic large cell lymphoma (sALCL).^6^ In 2013, ADCs achieved another breakthrough with the approval of Trastuzumab emtansine (TDM‐1) by the US FDA for human epidermal growth factor receptor‐2 (HER2)‐positive breast cancer.^7^ This marked the first ADC targeting solid tumors. Henceforth research on ADC drugs has rapidly advanced, leading to the emergence of a growing number of ADC drugs in the market.^8^ The evolution of ADC drugs from inception to maturity over the last century is depicted in Figure 1.

The research and development of antibody drugs have seen continuous advancements, leading to the classification of marketed ADC drugs into three generations.^9^ The first‐generation ADCs had limitations such as insufficient toxicity in the payload, unstable structure, and easy detachment of the toxin, resulting in a narrow therapeutic window and a high rate of failure.^4^ The second‐generation ADCs, utilizing humanized monoclonal antibodies and more potent cytotoxic drugs, showed reduced immunogenicity, improved drug efficacy, and a wider therapeutic window compared with the first generation.^10^, ^11^ The third‐generation ADC drugs introduced site‐specific conjugates, enhancing the uniformity of drug‐to‐antibody ratio (DAR), reducing toxic side effects, and increasing efficacy, thereby expanding the treatment window.^12^ Additionally, antibody optimization and new small molecules have improved drug specificity and therapeutic effects, with binding activity to cells expressing lower antigen levels.^13^

ADC drugs exhibit distinct pharmacological properties and show significant activity in the field of antitumor treatment, while maintaining a higher level of safety compared with traditional chemotherapy.^2^, ^14^, ^15^, ^16^ In addition to pursuing enhanced efficacy, the current focus of research and development in ADC drugs also involves addressing the issue of therapeutic resistance.^17^ This review aims to delve into the recent advancements in understanding the mechanisms of ADC action and resistance, as well as strategies to combat such resistance. The article further examines in detail the pivotal trials that contributed to the US FDA approval of all ADCs, with a particular focus on the robustness of the evidence. It also provides valuable insights into potential future research directions in this field.

## 2 MECHANISTIC INSIGHTS INTO ADCs IN TARGETED CANCER THERAPY

**FIGURE 2:**
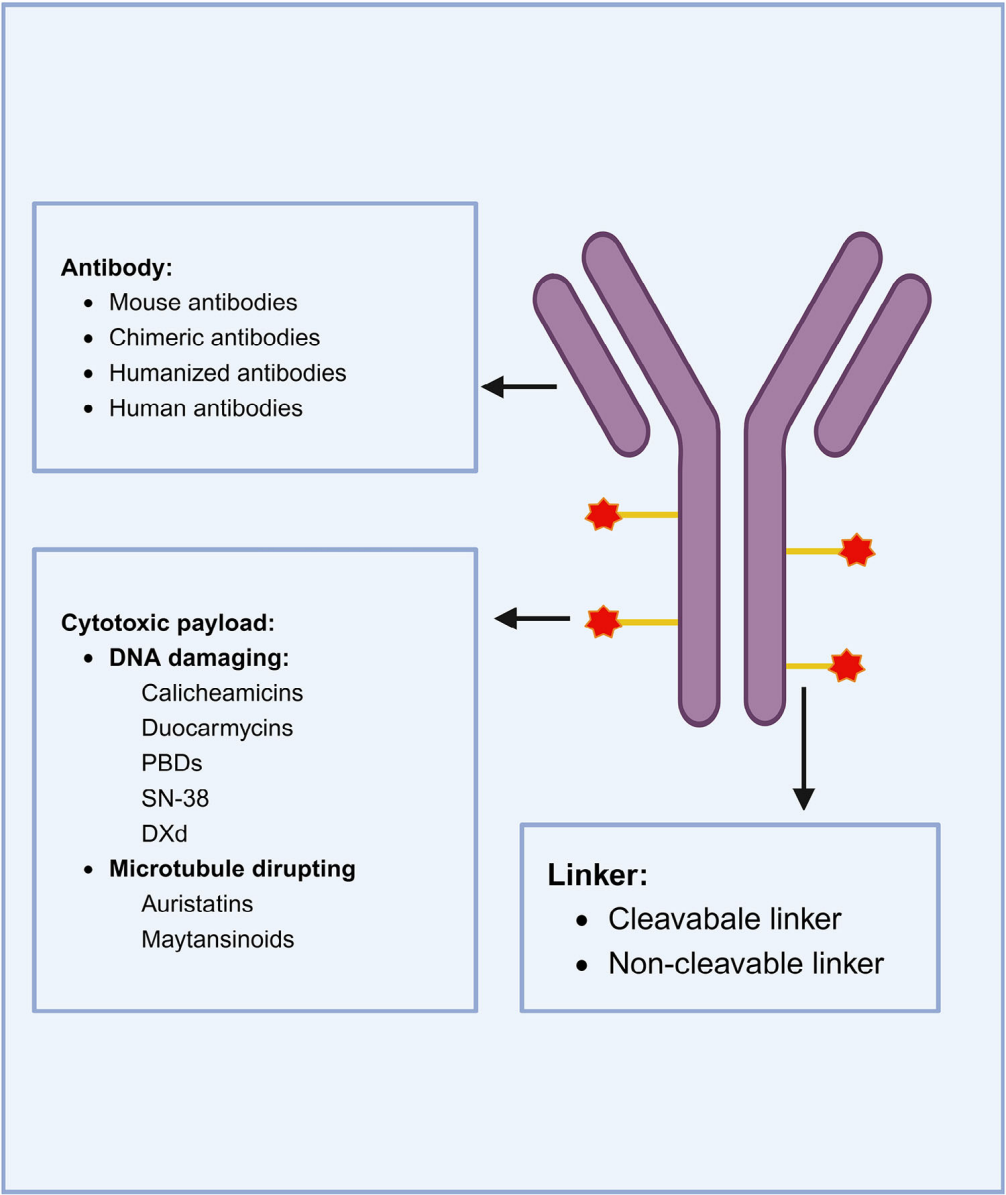
Structure of antibody–drug conjugates. The three essential components of an ADC include antibodies, cytotoxic payloads, and linkers. Antibodies are classified into four main types: mouse antibodies, chimeric antibodies, humanized antibodies, and human antibodies. Cytotoxic payloads can be categorized into two groups based on their mode of action: molecules that act on DNA, such as calicheamicins, duocarmycins, PBDs, SN‐38, and DXd, and molecules that act on tubulin (such as auristatins and maytansinoids). Linkers can be broadly categorized into two groups: cleavable and noncleavable. PBD, pyrrole benzodiazepines; SN‐38, 7‐ethyl‐10‐hydroxycamptothecin; DXd, deruxtecan.

**FIGURE 3:**
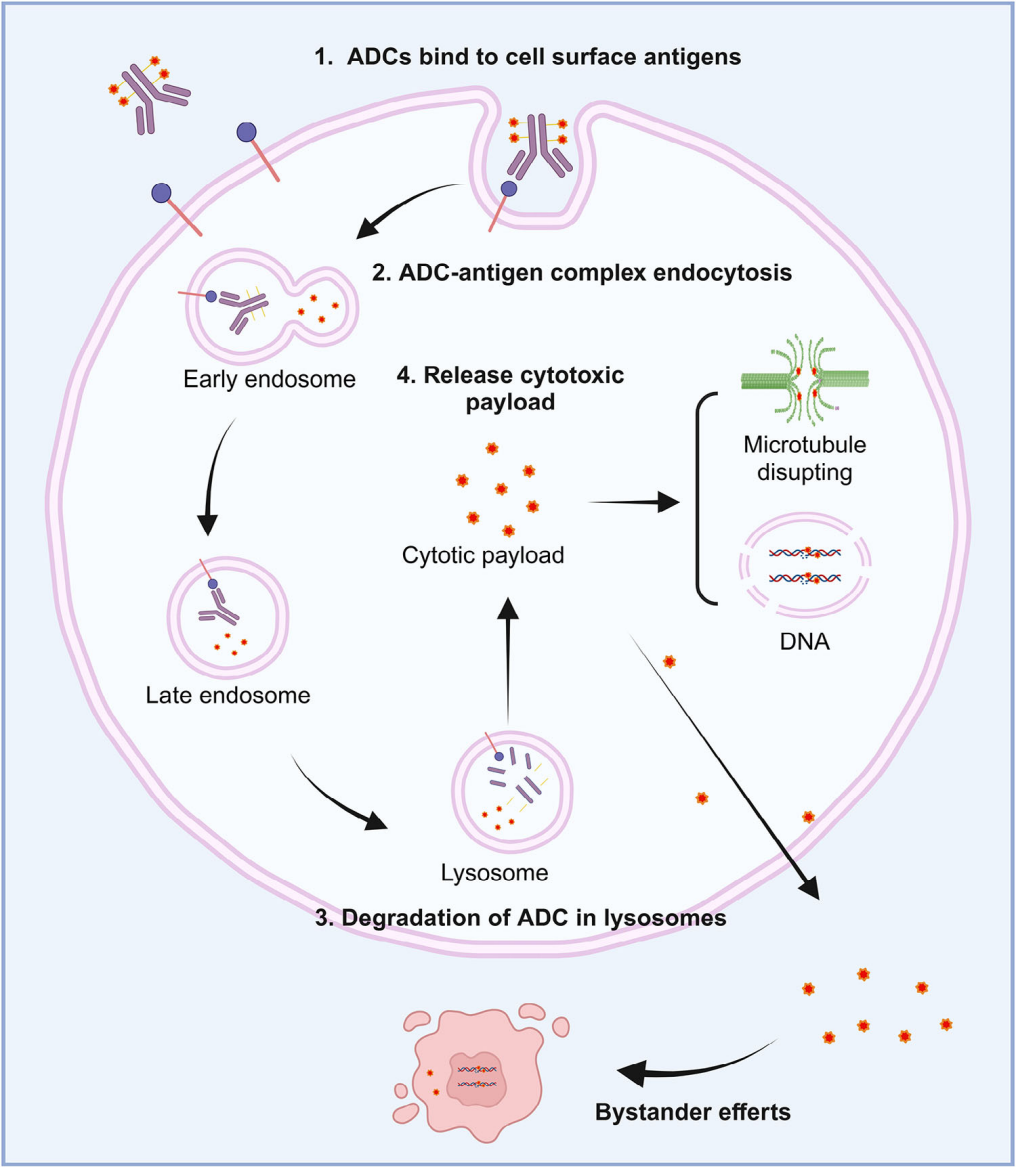
Action mechanism of ADCs. The antibodies bind to antigens on the cell surface (1) and form ADC–antigen complexes. These complexes are then taken into lysosomes through receptor‐mediated endocytosis (2). Within the lysosomes, the ADCs can be broken down by enzymes or the acidic environment (3). This breakdown releases cytotoxic payloads that can either damage DNA or inhibit microtubule polymerization (4).

ADCs are composed of an antibody connected to a cytotoxic drug through a linker (Figure 2). This unique structure offers ADC drugs multiple advantages, allowing them to combine the potent killing capabilities of traditional small molecule chemotherapy with the targeted delivery features of antibodies. Upon administration of ADCs, the antibody component binds to the target antigen on the surface of tumor cells. Subsequently, tumor cells uptake the ADC molecules. A fraction of the ADC can attach to Fc receptors in endosomes, aiding in its transport to the cell surface. Through FcRn‐mediated transcytosis, this fraction is then released outside the cell. Conversely, other ADC–antigen complexes enter lysosomes, where enzymes or acidic conditions can degrade the ADC. Consequently, cytotoxic agents are released, which can either harm DNA or impede the division of tumor cells, ultimately resulting in their demise (Figure 3). The mechanism of ADCs described above is intricately linked to specific targeting antibodies, potent cytotoxic drugs, the linker, and the target antigen.

### 2.1 Antibody

The antibody component of the ADC recognizes the target antigen, and in disease treatment, monoclonal antibodies are typically used for their ability to specifically recognize a single antigen.^18^, ^19^ This specificity enhances the targeting of the drug and reduces off‐target effects.

Monoclonal antibodies can be classified into four main types: mouse antibodies, chimeric antibodies, humanized antibodies, and human antibodies.^19^ Initially, mouse‐derived antibodies were commonly used, but they posed issues such as immunogenicity, poor efficacy, and short half‐life when introduced into the human body.^19^ In 1975, hybridoma technology enabled the development of chimeric antibodies, which have human constant regions and mouse variable regions.^20^ Chimeric antibodies reduce the mouse component while enhancing human properties, although immunogenicity remains a concern.^21^ To address this, researchers further minimized the mouse components to the complementarity‐determining regions, resulting in humanized antibodies.^19^ With the advancement of recombinant protein expression technology, fully human antibodies could be produced. Currently, the majority of ADCs on the market or under development utilize humanized or fully human antibodies, which exhibit strong antigen‐binding ability, high selectivity, long half‐life, and minimal immunogenicity.^22^, ^23^

Bispecific antibody ADC therapy has seen a recent surge in popularity.^24^, ^25^, ^26^, ^27^ By incorporating bispecific antibodies into ADCs, researchers aim to enhance the cellular internalization of ADCs. Bispecific ADCs can be strategically designed to bind to two distinct, nonoverlapping epitopes on the same target antigen, leading to the formation of robust receptor clusters and improved internalization, lysosomal transport, and degradation processes. Additionally, bispecific ADCs can be engineered to target different antigens. For instance, studies have demonstrated that bispecific ADCs targeting both HER2 and prolactin receptors (PRL‐R) exhibit superior efficacy in killing target cells expressing both HER2 and PRL‐R compared with ADCs targeting only HER2. This suggests that combining the ADC target (e.g., HER2) with a rapidly internalizing protein (e.g., PRL‐R) facilitates swift internalization and lysosomal degradation.^28^ Furthermore, the use of bispecific antibodies in ADCs can enhance the selectivity of tumor targeting by binding to two antigens on tumor cells. Despite the promising potential of bispecific antibody ADCs, this field is still in the early stages of research and development, with most products currently undergoing preclinical investigations.^25^

### 2.2 Effector molecule: cytotoxic payload

The cytotoxic payloads of ADCs act as the potent end‐effector components responsible for killing cancer cells. The toxicity of these effector molecules in ADCs is often higher than that of ordinary small molecules, a characteristic inherent to ADCs. Each antibody typically carries a limited number of effector molecules, known as the DAR value, which determines the pharmacokinetics, potency, and toxicity of the ADC drug. In traditional random drug conjugation strategies, drugs with varying DAR values are mixed together, and the DAR of each antibody may vary between 0 and 8.^29^, ^30^, ^31^ The heterogeneity of DAR will result in heterogeneity in pharmacokinetics, efficacy, and safety and will also lead to unstable drug structure, increased off‐target toxicity, drug aggregation, and other problems that are not conducive to later development.^32^, ^33^ Each antibody typically carries a limited number of effector molecules, resulting in heterogeneity that impacts pharmacokinetics, efficacy, safety, and overall drug stability.^31^, ^34^ While maximizing DAR can enhance antitumor efficacy, it is essential to balance this with concerns such as increased protein aggregation and ADC clearance that may reduce efficacy and safety. Strategies like site‐specific conjugation, involving engineered cysteine residues, unnatural amino acids in antibody sequences, and enzymatic conjugation through glycotransferases and transglutaminases, aim to make DAR more homogeneous.^35^, ^36^, ^37^ Maintaining a suitable DAR value remains a critical aspect that requires further exploration.

The cytotoxic molecules commonly used in cancer treatment are typically classified into three categories based on their mode of action.^38^, ^39^ The first category includes molecules that target DNA or topoisomerase I, such as calicheamicins, duocarmycins, pyrrolobenzodiazepines, SN‐38 (the active form of irinotecan), and deruxtecan. The second category consists of tubulin inhibitors used for payloads, like auristatins and eribulin from marine sources, and maytansinoids from plants. While other tubulin inhibitors, such as derivatives of taxol, vincristine, and colchicine, have been explored in ADCs, their efficacy has been limited.^40^, ^41^ The third category comprises alkylating agents, like duocarmazine.

Several dual‐payload ADC synthesis strategies have been developed to attach two drugs to the same linker.^42^, ^43^, ^44^, ^45^, ^46^ For example, Levengood *et al*.^43^ introduced the first dual‐loaded ADC that combines monomethyl auristatin E (MMAE) and monomethyl auristatin F (MMAF) on a short peptide ligand with orthogonally protected cysteine residues, resulting in a DAR of 16 (8+8). This dual‐loaded ADC exhibited potent antitumor activity, and resistance in tumor cells could potentially be overcome by adding complementary payloads.

Additionally, some ADC drugs kill cancer cells through “bystander effect.”^47^ Cleavable linkers are essential for inducing the bystander effect.^48^ ADCs containing cleavable linkers are internalized by tumor cells that have high antigen expression. Once inside the cells, the drugs degrade in lysosomes, releasing free toxins (hydrophobic and uncharged) that can either exit the cell membrane to directly kill target cells or penetrate the membrane to reach the tumor microenvironment. In the tumor microenvironment, they can attack surrounding tumor cells with low or no antigen expression (referred to as bystander cells), thus demonstrating the bystander effect.^47^, ^49^ However, it is important to note that cleavable linkers also pose potential safety risks.^48^ The bystander effect may lead to the diffusion of free toxins from target tumor cells or their entry into the bloodstream, potentially causing toxicity to nontumor tissues.^49^, ^50^

### 2.3 Linker

The linker is a crucial component in ADC design, facilitating the connection between the antibody and the cytotoxic payload through covalent coupling. While primarily serving to link the antibody and effector molecule, the linker plays a multifaceted role beyond mere connection.^51^ Various properties of the linker, such as conjugation chemistry, length, and steric hindrance, directly impact the toxicity, specificity, stability, and efficacy of ADCs.^52^ An optimal linker should exhibit stability in circulation (with a half‐life approximately 10 times longer than that of the ADC) and efficiently release the cytotoxic payload within the tumor post‐internalization.^53^, ^54^ Additionally, the hydrophilicity or lipophilicity of the linker can influence payload coupling and reduce immunogenicity.

Researchers have explored different types of linkers, which can be broadly classified into two categories: cleavable and noncleavable linkers.^51^, ^55^, ^56^ Cleavable linkers have the ability to release a metabolite of the effector molecules from the ADC, allowing it to enter cells, either at the tumor site or within endosomes and lysosomes. These cleavable linkers are further divided into chemically cleavable linkers and enzymatically cleavable linkers, such as peptide‐based linkers, β‐glucuronide‐based linkers, and phosphate‐based linkers.^51^ Notably, β‐glucuronide‐based linkers consist of β‐glucuronidase‐sensitive linkers and β‐galactosidase‐sensitive linkers. Premature cleavage of these linkers can lead to the effector molecules harming normal cells.^57^ On the other hand, noncleavable linkers, such as thioether linkers and maleimido caproyl linkers, require internalization of the ADC into cells and subsequent breakdown by lysosomes to release the effector molecules. ADCs with noncleavable linkers depend on complete lysosomal enzymatic degradation of the antibody for payload release, resulting in simultaneous dissociation of the linker.^58^ Therefore, the use of noncleavable linkers must ensure that the linker–drug complex retains the ability to kill tumor cells. Considering the cytotoxicity of the payload and bystander effect, cleavable linkers are prioritized in ADC development, despite a few approved noncleavable effective linkers.

In recent decades, significant progress has been made in optimizing the structure and expanding the mechanisms of ADCs. This includes the development of various cleavable linkers such as cathepsin‐cleavable, acid‐cleavable, glutathione‐cleavable, Fe(II)‐cleavable, novel enzyme‐cleavable, photoresponsive, and bioorthogonal linkers.^59^, ^60^, ^61^ These novel cleavable linkers have shown increased selectivity for targeting tumors. Among them, cathepsin, glutathione, and acid‐cleavable linkers have been extensively researched and incorporated into approved ADCs.^58^, ^62^ Notably, phosphatase‐responsive and bioorthogonal cleavable linkers have the potential to overcome intracellular drug release limitations seen in traditional ADCs.^51^, ^63^ Early data on these new linkers are promising and are expected to significantly advance the development of ADC drugs in the future.

### 2.4 Target antigen

In previous studies, various strategies have been devised to combat cancer, including neutralizing target receptors, downregulating receptor levels, interrupting cellular pathways, antibody‐dependent cell‐mediated cytotoxicity, complement‐dependent cytotoxicity, ADCs‐dependent cellular phagocytosis, and suppressing immune checkpoints.^64^, ^65^, ^66^ However, a different approach is taken by ADCs. This method involves initially binding the antibody moiety to the target antigen, which is then transported into the cell through receptor internalization, allowing the effector molecule to exert a cytotoxic effect.^8^ Therefore, the selection of the target antigen plays a crucial role in ADC.

One major drawback of small molecule drugs is their lack of specificity, leading to toxicity not only in cancer cells but also in normal cells. ADCs effectively overcome this issue by precisely delivering the effector molecules to cancer cells. As a result, the identification of specific antigens of cancer cells has become increasingly feasible since the discovery of the first tumor antigen in the 1970s,^20^ thereby accelerating the development of ADCs.

Among the ADCs used in the treatment of breast cancer, the most commonly targeted receptor for monoclonal antibodies is HER2.^67^, ^68^, ^69^ Around 90−95% of AML patients express the CD33 protein on the surface of leukemic blast cells, making it an ideal target for the treatment of this disease.^70^ The P97 receptor is highly expressed on the surface of melanoma cells, with each cell having a number ranging from 80,000 to 280,000, whereas its expression level on the surface of other tumor cells is very low.^71^ Consequently, the ADC drug L49‐vcMMAF can selectively act on melanoma cells without affecting other tumor cells.^71^ However, it is important to note that high expression of antigens alone does not guarantee the effectiveness of ADC drugs. For instance, although CD21 is highly expressed, it forms a complex with CD19 on the surface of B cells, which hinders its internalization.^72^ Consequently, ADC drugs targeting CD21 cannot effectively enter the cells to exert their action.^72^

A suitable target antigen for ADC therapy must meet the following criteria. First, the target antigen should be highly expressed on tumor cells and have low expression on normal cells. Second, while phage display techniques can be used to isolate the genes coding for target‐specific antibody variable regions, many antibodies used in ADCs are still produced by hybridoma technology. Therefore, the target molecule needs to be antigenic. Third, the target antigen should be present on the cell surface to enable interaction with ADCs in the external environment. Last, the target antigen should have the ability to internalize, allowing the bound ADC molecules to enter the cells. These conditions are crucial as the functionality of ADCs relies on them.^73^, ^74^, ^75^, ^76^ While there are more than 300 target antigens for antibody‐based therapy, only around 50 have been developed as ADC targets (Table 1). Of note, although most approved ADCs are targeting internalizing antigens, ADCs targeting noninternalizing antigens at the surface of cancer cells or the stroma can also be developed.^77^, ^78^

**TABLE 1:** Target antigens for ADCs in clinical development.

Disease	Target antigens
Acute myeloid leukemia	CD25, CD33, CD123 (IL‐3R*α*), FLT3
Non‐Hodgkin lymphoma	CD19, CD20, CD21, CD22, CD25, CD30, CD37, CD70, CD71 (transferrin R), CD72, CD79a/b, CD180, CD205 (Ly75), ROR1
Hodgkin's lymphoma	CD25, CD30, CD197 (CCR7)
Multiple myeloma	CD38, CD46 (MCP), CD56, CD74, CD138,CD269 (BCMA), endothelin B receptor
Breast cancer	CD25, CD174, CD197 (CCR7),CD205 (Ly75), CD228 (P79), c‐Met, CRIPTO, ErbB2 (HER2), ErbB3 (HER3), FLOR1 (FRα), Globo H, GPNMB, IGF‐1R, integrin β‐6, PTK7 (CCK4), Nectin‐4 (PVRL4), ROR2, SLC39A6 (LIV1A, ZIP6)
Gastric cancer	CD25, CD197 (CCR7), CD228 (P79, SEMF), FLOR1, Globo H, GRP20, GC‐C, SLC39A6 (LIV1A, ZIP6)
Colorectal cancer	CD74, CD174, CD166, CD227 (MUC1), CD326 (Epcam), CEACAM5, CRIPTO, FAP, ED‐B, ErbB3 (HER3)
Liver cancer	CD276 (B7‐H3), c‐Met
Pancreatic cancer	CD25, CD71 (transferrin R), CD74, CD227 (MUC1), CD228 (P79, SEMF), GRP20, GC‐C, IGF‐1R, integrin β‐6, Nectin‐4 (PVRL4), SLC34A2 (NaPi2b), SLC44A4, *α*v*β*6, mesothelin
Lung cancer	CD25, CD56, CD71 (transferrin R), CD228 (P79, SEMF), CD326, CRIPTO, EGFR, ErbB3 (HER3), FAP, Globo H, GD2, IGF‐1R, integrin β‐6, Mesothelin, PTK7 (CCK4), ROR2, SLC34A2 (NaPi2b), SLC39A6 (LIV1A ZIP6), Axl, *α*v*β*6
Renal cancer	AGS‐16, EGFR, c‐MET, CAIX, CD70, FLOR1 (FRα), TIM‐1
Bladder cancer	CD25, CD205 (Ly75)
Ovarian cancer	CA125 (MUC16), CD142 (TF), CD205 (Ly75), FLOR1 (FRα), Globo H, mesothelin, PTK7
Prostate cancer	CD46 (MCP), PSMA, STEAP‐1, SLC44A4, TENB2
Head and neck cancer	CD71 (transferrin R), CD197 (CCR7), EGFR, SLC39A6 (LIV1A ZIP6)
Melanoma	CD276 (B7‐H3), GD2, GPNMB, ED‐B, PMEL 17, endothelin B receptor
Gliomas	CD25, EGFR
Mesothelioma	Mesothelin, CD228 (P79, SEMF)

Abbreviations: BCMA, B cell maturation antigen; c‐MET, c‐mesenchymal–epithelial transition factor; CA 125, carbohydrate antigen125; CAIX, carbonic anhydrase 9; CCR7, chemokine receptor 7; CD, cluster of differentiation; CEACAM5, carcinoembryonic antigen‐related cell adhesion molecule‐5; ED‐B, extra‐domain B; EGFR, epidermal growth factor receptor; ErbB2, erythroblastic leukemia viral oncogene homolog 2; ErbB3, Erb b2 receptor tyrosine kinase 3; FAP, fibroblast activation protein‐α; FLT3, Fms‐like tyrosine kinase 3; GC‐C, guanylate cyclase‐C; Globo H, globohexaosylceramide; GPNMB, glycoprotein nonmetastatic B; GRP 20, glycine‐rich protein 20; Her2, human epidermal growth factor receptor 2; HER3: human epidemal growth factor receptor 3; IGF‐1R, insulin‐like growth factor 1 receptor; MCP: membrane cofactor protein; MUC 1, mucin 1; PSMA: prostate‐specific membrane antigen; PTK7, protein tyrosine kinase 7; PVRL4, poliovirus receptor‐like protein 4; STEAP‐1, six‐transmembrane epithelial antigen of prostate‐1; SLC44A4, solute carrier family 44 member 4; ROR1, receptor tyrosine kinase‐like orphan receptor 1; ROR2, receptor tyrosine kinase‐like orphan receptor 2; SLC39A6, solute carrier family 39 member 6; TF, tissue factor; TIM‐1, T cell immunoglobulin mucin domain 1.

## 3 THERAPEUTIC TRIALS

The technology for developing ADCs has been consistently advancing, resulting in a growing number of ADC drugs being developed for different types of cancer. Currently, over 300 ADC candidates are being studied at various clinical trial phases, leading to an increase in market share due to their encouraging outcomes.

The most popular research and development of ADCs focus on tumors. Additionally, ADC drugs can be effective in patients with low tumor antigen expression through their bystander effect, thus expanding the indication population. Clinical trials have also revealed the expansion of indications to include infections (e.g., human immunodeficiency virus, lung diseases), autoimmune diseases (such as spinal arthritis and Alzheimer's disease), and metabolic diseases (such as obesity and diabetes).^79^, ^80^, ^81^, ^82^, ^83^, ^84^

In the field of oncology, currently approved ADCs target specific proteins overexpressed by cancer cells, such as HER2, trophoblast cell surface antigen 2 (Trop2), Nectin‐4, and EGFR in solid tumors, and CD19, CD22, CD33, CD30, and CD79b in hematologic malignancies.^85^ The success of T‐DM1 and recent approvals like T‐Dxd, sacituzumab govitecan (SG), and enfortumab vedotin have led to an increase in ADCs for solid tumors.^17^, ^86^, ^87^, ^88^ Research in oncology and immunology has expanded the selection of ADC target antigens beyond traditional tumor cell antigens to include targets in the tumor microenvironment, like the stroma and vasculature.^89^, ^90^ Recent evidence from preclinical and clinical studies suggests that components of the neovascular system, subcutaneous extracellular matrix, and tumor matrix could serve as valuable target antigens for ADC drug development.^91^ Several key clinical studies that have resulted in US FDA‐approved ADCs are outlined in Table 2.

**TABLE 2:** Summary of ADCs approved by US FDA.

ADC	Target	Playload	Linker	DAR	Trials (NCT)	Phase	Main indication	Clinical registration website
Gemtuzumab ozogamicin	CD33	N‐acetyl calicheamicin	Cleavable hydrazone and disulfide linker	2–3	Approved again based on the results of ALFX‐0701 (NCT00927498), AML‐19 (NCT00091234), MyloFrance‐1	III, III, II	Acute myelocytic leukemia (2000, withdrawn 2010, approved again 2017)	https://clinicaltrials.gov/study/NCT00927498?term=nct00927498&rank=1; https://clinicaltrials.gov/study/NCT00091234?term=NCT00091234&rank=1; https://www.nature.com/articles/2404434
Brentuximab vedotin	CD30	MMAE	Cleavable valine–citrulline linker	4	(NCT01393717) and (NCT00848926), ECHELON‐2 (NCT01712490), ALCANZA (NCT01578499), (NCT01657331), AHOD1331 (NCT02166463)	II and II, III, III, I−II, III	Hodgkin lymphoma (2011), systemic anaplastic large cell lymphoma (2011), Mycosis fungoides (2017), CD30‐expressing lymphomas in combination with chemotherapy (2018), Hodgkin lymphoma in combination with chemotherapy in children (2022)	https://clinicaltrials.gov/study/NCT01393717?term=NCT01393717&rank=1; https://clinicaltrials.gov/study/NCT00848926?term=NCT00848926&rank=1; https://clinicaltrials.gov/study/NCT01712490?term=NCT01712490&rank=1; https://clinicaltrials.gov/study/NCT01578499?term=NCT01578499&rank=1; https://clinicaltrials.gov/study/NCT01657331?term=NCT01657331&rank=1; https://clinicaltrials.gov/study/NCT02166463?term=NCT02166463&rank=1
Trastuzumab emtansine	HER2	Maytansine derivative	Noncleavable linker	3.5	EMILIA (NCT00829166)	III	Breast cancer (2013)	https://clinicaltrials.gov/study/NCT00829166?term=NCT00829166&rank=1
Inotuzumab ozogamicin	CD22	N‐acetyl calicheamicin	Cleavable hydrazone and disulfide linker	6	INO‐VATE ALL (NCT01564784)	III	B‐acute lymphoblastic leukemia (2017)	https://clinicaltrials.gov/study/NCT01564784?term=NCT01564784&rank=1
Polatuzumab vedotin	CD79b	MMAE	Cleavable valine– citrulline linker	4	GO29365 (NCT02257567)	II	Diffuse large B cell lymphoma (2019)	https://clinicaltrials.gov/study/NCT02257567?term=NCT02257567&rank=1
Loncastuximabtesirine	CD19	PBD	Cleavable valine– alanine linker	2.3	LOTIS‐2 (NCT03589469)	II	Large B cell lymphoma (2021)	https://clinicaltrials.gov/study/NCT03589469?term=NCT03589469&rank=1
Belantamab mafodotin	CD269 (BCMA)	MMAF	Noncleavable linker	4	DREAMM‐2 (NCT03525678)	II	Multiple myeloma (2020) withdrawn in 2023 based the results of DREAMM‐3	https://clinicaltrials.gov/study/NCT03525678?term=NCT03525678&rank=1
Enfortumab vedotin	Nectin‐4	MMAE	Cleavable valine–alanine linker	3.8	EV‐201 trial (NCT03219333), EV‐302 trial (NCT04223856)	II, Ib/II	Urothelial carcinoma (2019); in combination with pembrolizumab for urothelial carcinoma (2023)	https://clinicaltrials.gov/study/NCT03219333?term=NCT03219333&rank=1; https://clinicaltrials.gov/study/NCT04223856?term=NCT04223856&rank=1
Trastuzumab emtansine	HER2	Maytansine derivative	Nonreducible thioether linker	3.5	KATHERINE trial (NCT01772472)	II	Breast cancer (2020)	https://clinicaltrials.gov/study/NCT01772472?term=NCT01772472&rank=1
Trastuzumab deruxtecan	HER2	Exatecan derivative	Cleavable glycine–glycine–phenylalanine–glycine linker	7.7	DESTINY‐Breast01 (NCT03248492), DS8201‐A‐J101 (NCT02564900), DESTINY‐Breast04 trial (NCT03734029)	II, I, III	HER2+ breast cancer (2019); HER2+ gastric cancer (2021); HER2 low breast cancer (2022)	https://clinicaltrials.gov/study/NCT03248492?term=NCT03248492&rank=1; https://clinicaltrials.gov/study/NCT02564900?term=NCT02564900&rank=1; https://clinicaltrials.gov/study/NCT03734029?term=NCT03734029&rank=1
Sacituzumab govitecan	TROP2	SN‐38	Cleavable lysine–PAB and carbonate linker	7.6	IMMU‐132‐01 trial (NCT01631552), TROPHY‐U‐01 (NCT03547973), ASCENT trial (NCT02574455)	I, II, III	Triple‐negative breast cancer (2020); Urothelial carcinoma (2021); HR+, HER2− breast cancer (2023)	https://clinicaltrials.gov/study/NCT01631552?term=NCT01631552&rank=1; https://clinicaltrials.gov/study/NCT03547973?term=NCT03547973&rank=1; https://clinicaltrials.gov/study/NCT02574455?term=NCT02574455&rank=1
Tisotumab vedotin	Tissue factor	MMAE	Cleavable valine–citrulline linker	4	InnovaTV 201 (NCT02001623)	I–II	Cervical cancer (2021)	https://clinicaltrials.gov/study/NCT02001623?term=NCT02001623&rank=1
Mirvetuximab soravtansine	Folate receptor‐α	Maytansine derivative	Cleavable and disulfide linker	3.5	SORAYA trial (NCT04296890)	III	Folate receptor‐α ovarian, fallopian tube and peritoneal cancers (2022)	https://clinicaltrials.gov/study/NCT04296890?term=NCT04296890&rank=1
Patritumab deruxtecan	HER3	Exatecan derivative	Tetrapeptide based cleavable linker	8	U31402‐A‐U102 trial (NCT03260491)	I	Non‐small‐cell lung cancer (2023)	https://clinicaltrials.gov/study/NCT03260491?term=NCT03260491&rank=1

Abbreviations: ADC, antibody–drug conjugate; DAR, drug–antibody ratio; HER, human epidermal growth factor receptor; HR, hormone receptor; MMAE, monomethyl auristatin E; MMAF, monomethyl auristatin F; PBD, pyrrolobenzodiazepine.

### 3.1 HER2

HER2/ERBB2/NEU, a member of the ErbB receptor tyrosine kinase family, plays a crucial role in cell growth, differentiation, and survival.^92^ Amplification of HER2 has been associated with poor prognosis in various tumor types such as advanced breast cancer, gastric cancers, colorectal and gastroesophageal junction adenocarcinomas, and non‐small‐cell lung cancer.^93^ HER2‐targeted ADCs are currently the standard therapy for solid tumors expressing or harboring mutations in HER2.^94^, ^95^, ^96^

TDM‐1 was the first ADC approved in 2013 for treating breast cancer. It combines the humanized monoclonal HER2‐targeting trastuzumab with the highly potent cytotoxic activity of the microtubule inhibitor DM1, a derivative of maytansine.^97^ Trastuzumab deruxtecan (DS‐8201; T‐DXd) is a novel humanized HER2‐directed ADC with a high DAR (8:1) that was approved by the US FDA in 2019.^98^ The structure of T‐DXd includes an anti‐HER2 IgG1 antibody (trastuzumab), a stable tetrapeptide‐based cleavable linker, and the exatecan derivative MAAA‐1181a (DXd), a DNA topoisomerase I inhibitor with 10‐fold higher inhibitory potency than irinotecan. T‐DXd has shown antitumor activity not only on HER2‐positive tumor cells but also on neighboring tumor cells with or without HER2 expression through a bystander effect.^98^ The efficacy of TDM1 and T‐DXd has been demonstrated in randomized trials as first‐line, second‐line, or later treatment for solid cancer. Disitamab vedotin (RC48) is composed of: (1) hertuzumab, a new generation anti‐HER2 humanized monoclonal antibody with high specificity and affinity for HER2; (2) a maleimide–cysteine–valine–citrulline–para‐aminobenzyloxycarbonyl linker that releases the cytotoxic payload; and (3) a cytotoxic payload, MMAE, which inhibits microtubule polymerization in actively dividing cells.^99^, ^100^ MMAE is a synthetic derivative of auristatin with potent antimitotic activity, leading to cell cycle arrest and eventual cell death.^101^

#### 3.1.1 Breast cancer

Phase III trials have been conducted to assess the efficacy of T‐DM1 in various treatment settings for HER2‐positive breast cancer, following promising results from phase I and II trials.^102^, ^103^ The EMILIA trial (NCT00829166) demonstrated that T‐DM1 improved progression‐free and overall survival (OS) compared with lapatinib plus capecitabine in patients with previously treated HER2‐positive metastatic breast cancer. Among 991 patients, T‐DM1 showed a median progression‐free survival (PFS) of 9.6 versus 6.4 months for lapatinib plus capecitabine, and a median OS of 30.9 versus 25.1 months, respectively. The rate of grade 3 or 4 adverse events was lower with T‐DM1 (41%) compared with lapatinib plus capecitabine (57%).

A subsequent open‐label phase III TH3RESA trial (NCT01419197) involved 602 HER2‐positive advanced breast cancer patients who had been previously treated with trastuzumab, lapatinib, and a taxane.^104^ The trial showed that the mOS was significantly longer with TDM‐1 compared with treatment of physician's choice (22.7 months [95% confidence interval (CI), 19.4–27.5] vs. 15.8 months [13.5–18.7]; HR 0.68 [95% CI, 0.54–0.85]; *p* = 0.0007). In the context of previously untreated HER2‐positive metastatic breast cancer, T‐DM1 demonstrated improved tolerability and noninferior PFS compared with a taxane plus trastuzumab in the phase III MARIANNE study (NCT01120184). However, T‐DM1 in combination with pertuzumab as neoadjuvant therapy resulted in a lower rate of pathological complete responses (CRs) when compared with the standard treatment of docetaxel, carboplatin, pertuzumab, and trastuzumab (44.4 vs. 55.7%, *p* = 0.016) as observed in the KRISTINE trial (NCT02131064).^105^, ^106^ Additionally, findings from the KATHERINE trial (NCT01772472) indicated that adjuvant treatment with T‐DM1 reduced the risk of recurrence of invasive disease or death by 50% compared with continuing adjuvant trastuzumab among patients with HER2‐positive early breast cancer and residual invasive disease after neoadjuvant chemotherapy plus HER2‐targeted therapy.^107^ Notably, patients receiving T‐DM1 experienced a higher percentage of grade ≥3 adverse events compared with those receiving trastuzumab (25.7 vs. 15.4%).^107^ Overall, based on these results, T‐DM1 has been recommended for adjuvant, first‐line, and second‐line treatment in breast cancer patients.^108^

T‐DXd was initially assessed in HER2‐positive metastatic breast cancer patients who had previously been treated with T‐DM1. The DESTINY‐Breast01 trial (NCT03248492) demonstrated sustained antitumor effects, with 112 out of 184 participants (60.9%) showing a response to therapy at the recommended dosage of 5.4 mg/kg. The median PFS was reported to be 16.4 months.^109^ Although 13.6% of patients experienced interstitial lung disease, leading to four deaths, the US FDA granted accelerated approval for T‐DXd in adults with unresectable or metastatic HER2‐positive breast cancer who had undergone two or more prior treatments.^110^ Subsequently, the DESTINY‐Breast 02 trial (NCT03523585) compared the efficacy and safety of T‐DXd versus physician's choice treatment in 608 HER2‐positive unresectable or metastatic breast cancer patients.^111^ Results indicated a median PFS of 17.8 months in the T‐DXd group versus 6.9 months in the physician's choice group (HR 0.36 [0.28−0.45]; *p* < 0.0001). Common adverse events in both groups included nausea, vomiting, hair loss, fatigue, and diarrhea, with T‐DXd‐associated interstitial lung disease occurring in 42 (10%) patients. Overall, the study reinforced the favorable therapeutic outcomes of T‐DXd in HER2‐positive metastatic breast cancer patients.

In the recent DESTINY‐Breast03 clinical trial (NCT03529110), T‐DXd demonstrated a significant improvement in OS and PFS when compared with T‐DM1. This led to a 36% reduction in the risk of death for patients with HER2‐positive metastatic breast cancer who had previously been treated with trastuzumab plus a taxane.^112^ The median PFS for T‐DXd was 28.8 months (95% CI, 22.4–37.9) compared with 6.8 months (95% CI, 5.6–8.2) for T‐DM1, with a hazard ratio (HR) of 0.33 (95% CI, 0.26−0.43; *p* < 0.0001). The median OS was not reached for T‐DXd (95% CI, 40.5 months—not estimable) and T‐DM1 (34.0 months—not estimable), with an HR of 0.64 (95% CI, 0.47–0.87; *p* = 0.0037). The percentage of patients alive at 12 months was 94.1% (T‐DXd) and 85.9% (T‐DM1), with an HR of 0.55 (95% CI, 0.36–0.86). The ORR was 79.7% (T‐DXd) and 34.2% (T‐DM1). The DESTINY‐Breast04 trial investigated the effects of T‐DXd in patients with HER2‐low metastatic breast cancer who had received one or two prior lines of chemotherapy. Among all patients, the median PFS was 9.9 months (T‐DXd) and 5.1 months (physician's choice standard chemotherapy), with an HR of 0.50 (*p* < 0.001), and OS was 23.4 months (T‐DXd) and 16.8 months (standard chemotherapy), with an HR of 0.64 (*p* = 0.001). In the hormone receptor‐positive subgroup, the median PFS was 10.1 months (T‐DXd) and 5.4 months (physician's choice), with an HR of 0.51 (*p* < 0.001), and OS was 23.9 months (T‐DXd) and 17.5 months (physician's choice), with a HR for death of 0.64 (*p* = 0.003).

On the basis of these findings, T‐DXd significantly extends the survival of patients with HER2‐low expressing or HER2‐positive metastatic breast cancer, indicating its potential as an alternative treatment option for this specific population.^113^, ^114^ However, the confirmed objective response rate (ORR; primary endpoint) of T‐DXd in the phase II DAISY trial (NCT04132960) was 70.6% (95% CI, 58.3–81) in the HER2‐overexpressing group, 37.5% (95% CI, 26.4–49.7) in the HER2‐low expression group, and 29.7% (95% CI, 15.9–47) in the HER2 nonexpressing group.^115^ These results indicate that HER2 status remains a key factor in determining the responsiveness to T‐DXd.

#### 3.1.2 Non‐small cell lung cancer

In a phase II study by Hotta *et al*.,^116^ DM1 (at a dose of 3.6 mg/kg) demonstrated limited efficacy against HER2‐positive non‐small cell lung cancer (NSCLC) patients. The primary endpoint, an ORR, was achieved by only one patient (6.7%; 95% CI, 0.3–27.9), with seven patients (46.7%) showing stable disease (SD) and progressive disease (PD). The median PFS was 2.0 months (95% CI, 1.4–4.0), and the median OS was 10.9 months (95% CI, 4.4–12.0). In a separate phase II clinical study, T‐DM1 was administered to forty‐nine previously treated patients with advanced HER2‐overexpressing NSCLC (IHC 2+, 29; IHC 3+, 20) to evaluate its efficacy.^117^ The HER2 2+ cohort did not show any objective responses, with 28% having SD and 55% showing PD. In contrast, the HER2 3+ cohort had four partial responses (PRs) (overall response rate, 20%; 95% CI, 5.7–43.7). Clinical benefit rates were 7% (HER2 2+) and 30% (HER2 3+). Median OS was 12.2 months (95% CI, 3.8–23.3) in the HER2 2+ group and 15.3 months (95% CI, 4.1–not reached) in the HER2 3+ group, with comparable median PFS of 2.6 months (HER2 2+) and 2.7 months (HER2 3+).

The anticancer activity of T‐DXd in patients with HER2‐mutant NSCLC was initially studied in a dose‐expansion, phase I trial across various advanced solid tumors by Tsurutani *et al*.^118^ (NCT02564900). Among 11 previously treated NSCLC patients with HER2‐mutant disease, the median PFS was 11.3 months (95% CI, 8.1–14.3) with an ORR of 72.7% (eight out of 11). In another multicenter clinical trial, DESTINY‐Lung01 (NCT03505710), 91 patients with HER2‐mutant NSCLC were enrolled.^119^ The T‐DXd group showed a median PFS and OS of 8.2 months (95% CI, 6.0–11.9) and 17.8 months (95% CI, 13.8–22.1), respectively. However, 46% of patients experienced grade 3 or higher adverse events, with neutropenia (19%) being the most common. The randomized phase II trial, DESTINY‐Lung02 (NCT04644237), included 102 and 50 patients with HER2‐mutant metastatic NSCLC in the T‐DXd 5.4 and 6.4 mg/kg arms, respectively.^120^ Results showed an ORR of 49.0% (95% CI, 39.0–59.1) in the 5.4 mg/kg arm, and 56.0% (95% CI, 41.3–70.0%) in the 6.4 mg/kg arm. The median PFS and OS were 9.9 months (95% CI, 7.4–not reached) and 19.5 months (95% CI, 13.6–not reached) in the 5.4 mg/kg arm, and 15.4 months (95% CI, 8.3–not reached) and not reached (95% CI, 12.1–not reached) in the 6.4 mg/kg arm. The estimated 1‐year OS rate with T‐DXd 5.4 and 6.4 mg/kg was 67% (95% CI, 56–76) and 73% (95% CI, 57–84), respectively. Drug‐induced interstitial lung disease occurred in 28.0% of patients in the 6.4 mg/kg arm, compared with 12.9% in the 5.4 mg/kg arm. The US FDA has approved T‐DXd as the first anti‐HER2 agent for patients with HER2‐mutant NSCLC.^86^

#### 3.1.3 Gastric/gastroesophageal junction carcinomas

The DS8201‐A‐J101 (NCT02564900) trial was conducted to evaluate the safety, efficacy, and pharmacokinetics of T‐DXd in patients with advanced HER2‐positive gastric or gastroesophageal and breast cancer that had not responded to standard treatments.^121^ Results showed that all four patients with HER2‐positive gastric cancer achieved disease control, with two patients showing PR. A subsequent phase I study (NCT02564900) involving 44 previously treated patients with HER2‐positive gastric or gastroesophageal junction carcinomas demonstrated a manageable safety profile and promising antitumor activity of T‐DXd.^122^ Notably, 25% of patients experienced serious adverse events, with an ORR of 43.2%. A phase II trial comparing T‐DXd with chemotherapy in 125 patients with HER2‐positive advanced gastric cancer (vs. 62 patients receiving chemotherapy) revealed a significantly higher ORR with T‐DXd (51 vs. 14%, *p* < 0.001). Additionally, T‐DXd treatment led to a higher CR rate, greater reduction in tumor size, and longer mOS compared with chemotherapy. The median PFS was also longer in the T‐DXd group than in the chemotherapy group. The efficacy and safety of T‐DXd in HER2‐low gastric or gastroesophageal junction adenocarcinoma were investigated in an exploratory cohort study as part of a phase II trial. The results showed that T‐DXd demonstrated clinical activity in patients with heavily pretreated HER2‐low gastric/gastroesophageal junction adenocarcinoma.^123^ Among 19 patients with IHC 2+/ISH−, 26.3% achieved PR and 63.2% had SD. The median PFS and OS were 4.4 and 7.8 months, respectively, with a 12‐month OS rate of 40.0%. In the other cohort of 21 patients with IHC 1+, the disease control rate (DCR) was 71.4%, with 9.5% achieving PR and 61.9% having SD. The median PFS and OS were 8.5 and 2.8 months, respectively, with a 12‐month OS rate of 25.7%.

Patients with locally advanced or metastatic HER2‐positive gastric cancer or gastroesophageal junction adenocarcinoma, who had previously undergone trastuzumab‐based therapy, were granted US FDA approval to receive T‐DXd on January 15, 2021.^124^ Additionally, ongoing clinical trials are exploring the use of T‐DXd in HER2‐positive gastric cancer, both as monotherapy and in combination with chemotherapy and trastuzumab.

Disitamab vedotin has been approved in China for patients with HER2‐positive gastric cancer or gastroesophageal junction adenocarcinoma who have undergone two or more chemotherapy regimens based on several clinical trials.^100^, ^125^, ^126^, ^127^ The drug demonstrated significant efficacy, with an ORR of 15.0% and DCR of 45.0% in the initial trial (NCT02881190). In the analysis of gastric cancer patients with different HER2 statuses, the ORRs were 35.7% for IHC 2+/FISH−, 20% for IHC 2+/FISH+, and 13.6% for IHC 3+. In a subsequent phase II study (NCT03556345), 125 HER2‐positive patients with locally advanced or metastatic gastric or gastroesophageal junction cancer, who were on second‐line or later treatment, were included in the final analysis.^126^ The ORR and DCR, as assessed by an independent review committee, were 24.8% (95% CI, 17.5−33.3%) and 42.4% (95% CI, 33.6−51.6%), respectively. The 12‐month survival rate was 33.3%, with a median OS of 7.9 months (95% CI, 6.7–9.9). Combining disitamab vedotin with toripalimab showed enhanced clinical benefits, with a median PFS of 6.2 months (95% CI, 4.0–6.9%) and median OS of 16.8 months (95% CI, 7.2–not reached). PRs were observed in 12 out of 28 evaluable participants.^127^

#### 3.1.4 Other solid tumors

The HERACLES‐B (NCT03225937) clinical trial was the first to explore the use of ADC in patients with RAS/BRAF wild‐type and HER2+ metastatic colorectal cancer that was resistant to chemotherapy.^128^ In this phase II trial with 31 previously treated patients, pertuzumab was administered in combination with T‐DM1. While the primary objective of ORR was 9.7% (95% CI, 0–28%), falling below the expected success threshold, the DCR was 77.4% and PFS was 4.1 months (95% CI, 3.6–5.9). These results suggest that T‐DM1 could be a promising treatment option for HER2+ colorectal cancer.

Several clinical trials have shown promising efficacy and safety data in combining T‐DXd with other treatments for pretreated HER2‐expressing metastatic colorectal cancer.^129^, ^130^ The DESTINY‐CRC01 study (NCT03384940) enrolled 53 patients with HER2‐positive expression between February 2018 and July 2019, all of whom received T‐DXd. A confirmed objective response was seen in 24 patients (45.3%) during a follow‐up of over 27 weeks. The 6‐month PFS and OS rates were 53.0% (95% CI, 37.0–66.7) and 76.6% (95% CI, 61.5–86.4), respectively. Median PFS was 6.9 months (4.1–not reached), while median OS was not reached (95% CI, 0.74 months—not evaluable). Grade 3 or higher side effects were observed in at least 10% of patients, including decreased neutrophil count in 17 patients and anemia in 11 patients. However, T‐DXd did not show antitumor activity in patients with HER2‐low metastatic colorectal cancer tumors (IHC2^+^ and ISH‐negative; IHC1^+^), as no confirmed objective responses were noted.

The phase II study (KAMELEON, NCT02999672) provides support for the potential use of T‐DM1 monotherapy as a treatment option regardless of tumor type for patients with HER2‐positive advanced urothelial bladder cancer or pancreatic cancer/cholangiocarcinoma.^131^ PR was observed in five patients in the urothelial bladder cancer cohort and one patient in the pancreatic cancer/cholangiocarcinoma cohort, resulting in an ORR of 38.5 and 14.3%, respectively. None of the patients in either cohort achieved a CR.

The DESTINY‐PanTumor02 phase II trial assessed the effectiveness and safety of T‐DXd in patients with HER2‐expressing solid tumors (NCT04482309).^132^ A total of 268 eligible patients with HER2‐positive solid tumors were included, such as those with locally advanced, unresectable, or metastatic biliary tract, bladder, cervical, endometrial, ovarian, pancreatic, and salivary gland cancer, as well as other solid cancers like malignant neoplasm of unknown primary site, extramammary Paget disease, oropharyngeal neoplasm, cutaneous melanoma, and various others. Among the 267 patients evaluated, 99 patients (37.1%) showed a confirmed objective response according to investigator assessment. Notably, responses were seen in patients who had or had not received prior HER2 therapy. Overall, 100 patients (37.5% [95% CI, 31.6–43.6]) evaluated by independent central review achieved a confirmed ORR, with a median PFS of 6.9 months and OS of 13.4 months. Patients with IHC 3+ status had the most significant benefit from T‐DXd, with an ORR of 61.3%, median duration of response (DoR) of 22.1 months, median PFS of 11.9 months, and median OS of 21.1 months. These findings led to the US FDA granting accelerated approval to T‐DXd for HER2‐overexpressing (IHC 3+) solid tumors without alternative treatment options on April 5, 2024.

In a phase I trial (NCT04280341) conducted in China, 56 patients with solid tumors, including gastric, gastroesophageal junction, breast cancer, and other solid tumors, were treated with disitamab vedotin and toripalimab. Of the 24 patients who received disitamab vedotin, the confirmed ORR was 25% (95% CI, 10−47), DCR was 75% (95% CI, 53−90), and median OS was 10.5 months.^127^

### 3.2 TROP2

TROP2, a product of the tumor‐associated calcium signal transducer 2 (TACSTD2) gene, is a transmembrane glycoprotein with a short cytoplasmic tail, a single transmembrane region, and a large extracellular region. While sporadically expressed in normal tissue, TROP2 is frequently found in many tumors, playing a role in cancer cell proliferation, apoptosis, and invasion, often linked to poor prognosis.^133^, ^134^, ^135^, ^136^

Based on hRS7, a humanized IgG1 mAb targeting TROP2, a novel third‐generation ADC named SG was developed by conjugating hRS7 with the derivative CL2A of the metabolite of the topoisomerase inhibitor irinotecan. SG (IMMU‐132) demonstrated preliminary antitumor activity and acceptable tolerability in previously treated solid cancers during the phase I/II IMMU‐132‐01 basket trial (NCT01631552).^137^, ^138^, ^139^ The efficacy of SG was observed across various malignancies, with promising results seen in histological cohorts of NSCLC, small cell lung cancer (SCLC), urothelial carcinoma, and metastatic breast cancer.^140^, ^141^, ^142^, ^143^ Additionally, other anti‐TROP‐2 ADCs such as SKB264 and datopotamab deruxtecan have shown potent antitumor activity in preclinical studies.^144^, ^145^ Datopotamab deruxtecan, composed of a recombinant humanized anti‐TROP2 IgG1 mAb conjugated with a Topo I inhibitor (DXd) via a tetrapeptide‐based linker, is a TROP‐2 ADC with a cleavable tetrapeptide linker and a more potent topoisomerase inhibitor payload compared with SG.^145^ This design confers increased stability in circulation and a longer half‐life compared with SG.

SG has shown significant efficacy in treating relapsed patients with metastatic triple‐negative breast cancer (mTNBC).^146^, ^147^ The ASCENT clinical trials (NCT02574455) demonstrated that SG provided a statistically significant PFS and OS benefit compared with chemotherapy in mTNBC patients who had undergone at least two prior therapies. The ORR was 35% with SG, significantly higher than the 5% seen with single‐agent chemotherapy. Common side effects included myelosuppression and diarrhea. SG is the first ADC drug approved for mTNBC therapy targeting Trop‐2.^147^, ^148^ In patients with hormone receptor‐positive/HER2‐negative endocrine‐resistant metastatic breast cancer, SG showed a longer PFS compared with chemotherapy [5.5 months (95% CI, 4.2–7.0) vs. 4.0 months (95% CI, 3.1–4.4); HR 0.66, *p* = 0.0003)].^149^ In the TROPHY‐U‐01 phase II trial (NCT03547973), SG was effective in patients with metastatic urothelial carcinoma who had progressed on two prior lines of therapy,^142^, ^150^ with an ORR of 27% (95% CI, 19.5–36.6) at a median follow‐up of 9.1 months, and promising median PFS (5.4 months, 95% CI, 3.5–7.2 months) and OS (10.9 months, 95% CI, 9.0–13.8 months), respectively. These results led to US FDA approval of SG as a treatment option for patients with locally advanced or metastatic urothelial cancer who had previously received platinum‐based chemotherapy or immune checkpoint inhibitors. In the IMMU‐132‐01 trial, out of three lung cancer patients, one patient with SCLC (TROP‐2 expression by IHC of 3+) who had received two lines of chemotherapy, showed a 38% reduction in the sum of the longest diameters (NCT01631552). However, the other two patients with SCLC and NSCLC did not exhibit any antitumor activity.^139^ In a single‐arm expansion trial involving patients with metastatic NSCLC who had received multiple prior therapies, the ORR was 19% with a median PFS of 5.2 months (95% CI, 3.2–7.1 months) and a clinical benefit rate of 43%.^140^ Despite Trop‐2 being the target of SG, no correlation was found between TROP2 expression and responses.^140^ Subsequent studies demonstrated the efficacy and safety of SG in previously treated metastatic SCLC patients.^141^ Among 50 patients who had received prior therapies, seven had PR and twenty‐one had SD, resulting in an overall ORR of 14% and DCR of 56%. The median PFS and OS were 3.7 and 7.5 months, respectively. The clinical benefit rate (≥4 months) was estimated to be 34%. Notably, PR, PFS, and clinical benefit rate with SG were all enhanced in the second‐line therapy for patients who were responsive to previous treatments. It is worth mentioning that Trop‐2 tumor staining was not a requirement for patient selection in this study. The most common grade ≥3 adverse events included neutropenia (34%), fatigue (13%), diarrhea (9%), and anemia (6%).

The results of the phase III TROPION‐Breast01 study will help define the role of Dato‐DXd in the metastatic HR+/HER2‐ breast cancer setting in patients who have progressed on, or are ineligible for, endocrine therapy and after chemotherapy. Previous studies on Dato‐DXd have shown promising antitumor activity and manageable safety profiles in patients with advanced NSCLC (NCT03401385).^151^, ^152^ The ORR was 26%, with a median DoR of 10.5 months at the recommended dose of 6 mg/kg. Median PFS and OS were 6.9 and 11.4 months, respectively, regardless of TROP2 level. The clinical benefit and safety profile of Dato‐DXd have been evaluated in various clinical trials, supporting its potential benefit in patients with solid tumors such as NSCLC and breast cancer.

### 3.3 HER3

HER3/ErbB3 is a member of the EGFR/HER family of receptor tyrosine kinases, alongside EGFR (ErbB1 or HER1), HER2 (ErbB2), and HER4 (ErbB4).^153^ Unlike other members, HER3 has minimal intracellular tyrosine kinase activity, which is significantly weaker than that of fully activated EGFR.^154^ Its ligands, neuregulin 1 (NRG‐1) and neuregulin 2 (NRG‐2), trigger HER3 to form heterodimers with other receptors, such as EGFR and HER2, leading to downstream C‐terminal phosphorylation.^155^, ^156^ Additionally, HER3 can dimerize with non‐EGFR family receptors like the MET factor receptor and FGFR2.^157^, ^158^ Several tyrosine phosphorylation sites on HER3 directly bind to PI3K, thereby activating the PI3K/AKT signaling pathway crucial for cancer cell survival. HER3 also triggers other downstream pathways like MEK/mitogen‐activated protein kinase (MAPK), Jak/Stat, and c‐Src, promoting cell proliferation.^159^, ^160^ High HER3 expression has been linked to disease progression and reduced survival in cancer patients, making it a potential target for therapy.^161^, ^162^

Patritumab deruxtecan (HER3‐DXd) is an ADC developed by Daiichi Sanko targeting HER3.^163^, ^164^ The antibody component, patritumab (U3‐1287), specifically targets the extracellular domain of HER3, effectively inhibiting the formation of HER2/HER3 heterodimers. Both HER3‐DXd and DS‐8201 contain deruxtecan, a topoisomerase I inhibitor, attached to the antibody through a maleimide–GGFG junction at a cysteine site with a DAR of 8.0.^165^ A stable tetrapeptide‐based cleavable linker is enzymatically cleaved in tumor lysosomes, releasing DXd that can enter cells via a bystander effect.

The phase I U31402‐A‐U102 study (NCT03260491) assessed the efficacy of patritumab deruxtecan in patients with previously treated metastatic or unresectable NSCLC.^166^ The dose‐escalation phase focused on patients with EGFR‐mutant disease who had progressed after osimertinib or those without T790M mutations who had progressed after erlotinib, gefitinib, or afatinib. The primary goals were to determine the safety, tolerability, and recommended dose for expansion. Results indicated that at a dose of 5.6 mg/kg, patritumab deruxtecan achieved a DCR of 72% in 57 patients who had received TKIs and platinum‐based chemotherapy, with a median PFS of 8.2 months. The agent also demonstrated efficacy in patients previously treated with osimertinib and platinum‐based chemotherapy, showing a confirmed ORR of 39% and a DCR of 68%, with a median PFS of 8.2 months. Notable responses were observed in patients with brain metastases, with a confirmed ORR of 32% and a median PFS of 8.2 months, as well as in those without brain metastases, with a confirmed ORR of 41% and a median PFS of 8.3 months. In terms of safety, all patients receiving patritumab deruxtecan at the 5.6 mg/kg dose experienced treatment‐emergent adverse effects, with a small percentage leading to treatment discontinuation or dose adjustments, but no fatal side effects were reported.

A phase II trial (HERTHENA‐Lung01, NCT05338970) assessed the effectiveness of patritumab deruxtecan in a group of heavily treated patients with advanced NSCLC who had previously received EGFR tyrosine kinase inhibitor therapy and platinum‐based chemotherapy.^167^, ^168^, ^169^ The primary objective, ORR, was determined to be 29.8% (95% CI, 23.9–36.2), with median PFS and OS of 5.5 and 11.9 months, respectively. There was no significant difference in treatment outcomes observed between patients who had previously received osimertinib and chemotherapy. In patients with nonirradiated brain metastases, approximately one‐third had their brain metastases controlled, with an ORR of 33.3% (95% CI, 17.3–52.8). A subsequent phase III study (HERTHENA‐Lung02, NCT05338970) is currently ongoing to evaluate the safety and efficacy of patritumab deruxtecan in patients who have experienced disease progression after third‐generation EGFR TKI treatment.^170^

Seventy‐seven patients with previously untreated HR+/HER2− breast cancer were assessed for efficacy in the SOLTI‐1805 TOT‐HER3 Study (NCT04610528).^171^, ^172^ A significant change was noted in CelTIL score [= −0.8 × tumor cellularity (in %) + 1.3 × tumor‐infiltrating lymphocytes (in %)], with a median increase from baseline of 3.5 (interquartile range, −3.8 to 12.7; *p* = 0.003). An ORR of 45% was achieved, with responders showing a higher increase in CelTIL score compared with nonresponders (mean difference, +11.9 vs. +1.9). One hundred eighty‐two patients with HER3‐expressing advanced breast cancer received ≥1 dose of HER3‐DXd in a multicenter, phase I/II trial (NCT02980341).^173^ HER3‐low was defined as an IHC score of 1+, while HER3‐high was defined as an IHC score of 2+ or 3+. Objective responses were observed regardless of HER3 expression levels. Efficacy outcomes were reported for specific clinical subgroups: HR+/HER2− (ORR, 30.1%; median PFS, 7.4 months), TNBC (ORR, 22.6%; mPFS, 5.5 months), and HER2+ (ORR, 42.9%; mPFS, 11.0 months). HER3‐DXd exhibited comparable antitumor activity to SG in patients with advanced breast cancer, with a low rate of treatment‐related adverse reactions leading to discontinuation (9.9%).

### 3.4 EGFR

EGFR (HER1 or ERBB1) is a member of the ErbB family of tyrosine kinase receptors, playing essential roles in regulating cell proliferation, differentiation, migration, and survival.^174^, ^175^ Overexpression or mutation of EGFR has been linked to the development of various solid tumors, such as NSCLC, nasopharyngeal carcinoma, squamous cell carcinomas of the head and neck, and colorectal cancer.^176^ In addition to tyrosine kinase inhibitors for EGFR‐mutated non‐small cell lung carcinoma, ADCs targeting EGFR have recently emerged as promising therapeutics for patients with HER‐positive cancers.^177^, ^178^, ^179^

ABT‐414 was created through the conjugation of MMAF, which inhibits microtubule assembly, to the interchain cysteines of the EGFR‐specific humanized antibody (ABT‐806) using a noncleavable maleimidocaproyl linker, resulting in an average DAR of approximately 4.^180^ Results from a multicenter, phase I international study (NCT01800695) demonstrated preliminary efficacy and a manageable safety profile in patients with EGFR‐overexpressing recurrent glioblastoma. Among the 66 patients, the ORR was 6.8%, with a 6‐month PFS rate of 28.8% and a 6‐month OS rate of 72.5%.^181^ The most common adverse events reported were related to the eyes (91%), including blurred vision (65%), dry eye (29%), keratitis (27%), and photophobia (27%).

MRG003 is an EGFR‐targeted ADC composed of a humanized IgG1 monoclonal antibody linked to MMAE through a valine–citrulline linker.^182^ This ADC can specifically bind to EGFR on tumor cells, enter the cells through endocytosis, travel to the lysosome, and release MMAE via protease degradation. MMAE inhibits tubulin polymerization, halting mitosis, ultimately leading to the inhibition of tumor cell growth and inducing cell death. In a first‐in‐human dose‐escalation study (CTR20180310) involving patients with relapsed/refractory solid tumors, the DCR was 100% for EGFR‐positive patients receiving doses of MRG003 at or above 1.5 mg/kg.^182^ In a subset analysis from another phase I study (NCT04868344) involving 39 patients with refractory advanced squamous cell carcinomas of the head and neck, colorectal cancer, and nasopharyngeal carcinoma, all patients were found to be EGFR positive.^183^ The ORR was 20.5% with 8 PRs, while the DCR was 51.3% with 12 SDs. The median PFS for all patients was 2.8 months. Importantly, a positive association was observed between EGFR expression levels and the clinical outcomes observed with MRG003 treatment.

### 3.5 c‐Met

The cell‐surface receptor tyrosine kinase c‐Met undergoes a translation process resulting in a precursor protein that is posttranslationally modified into a three‐dimensional structure connected by disulfide bonds.^184^ The mature c‐Met comprises a 50 kDa extracellular α chain and a 140 kDa transmembrane β chain. c‐Met, along with its sole ligand hepatocyte growth factor (HGF), plays a crucial role in various cellular processes such as proliferation, survival, invasion, tissue development, and organ regeneration.^185^, ^186^ HGF exists initially as an inactive precursor form that is activated by serine proteases to become mature. The active HGF ligands include α chains with N‐terminal and Kringle domains, as well as β chains resembling a serine protease domain.^187^ The N‐terminal domain and K1 region of the HGF molecule bind strongly to c‐Met, creating a new binding site on the HGF‐β chain. This tight binding forms a complex between HGF and c‐Met, initiating signal transduction. The formation of this active complex leads to receptor polymerization, endocytosis, and phosphorylation of multiple tyrosine residues in the intracellular kinase domain, thereby activating various signaling pathways (JAK/STAT3, PI3K/Akt/NF‐κB, and Ras/Raf pathways) associated with tumor development, invasion, and metastasis.^188^, ^189^, ^190^

Telisotuzumab vedotin (Teliso‐V) is a novel c‐Met–targeted ADC consisting of a humanized monoclonal antibody, ABT‐700, combined with MMAE through a cleavable valine–citrulline peptide linker (ABT‐700–vc‐MMAE). This combination results in approximately three molecules of monomethyl auristatin per antibody. Teliso‐V is capable of internalization and has been demonstrated to release MMAE, which subsequently binds to tubulin, leading to cell death by inhibiting mitosis. In essence, Teliso‐V can effectively deliver chemotherapy drugs specifically to the interior of cancer cells expressing c‐Met using antibodies, thereby directly targeting and eliminating cancer cells. Previous studies have indicated that Teliso‐V exhibits antitumor activity in MET‐amplified and c‐Met‐overexpressing tumors due to its specific and high‐affinity binding.^191^

The initial human trial of Teliso‐V in patients with solid tumors was carried out by Strickler *et al*. in 2018 (NCT02099058).^192^ Among the 16 patients with c‐Met‐mutated NSCLC who received Teliso‐V (2.4–3.0 mg/kg), three patients (18.8%) achieved a PR with a median response duration of 4.8 months and a mPFS of 5.7 months. The majority of patients did not respond to Teliso‐V. The most common grade ≥3 adverse events associated with Teliso‐V were hypoalbuminemia, fatigue, neutropenia, and anemia (4% each). In a phase I study of Teliso‐V monotherapy for patients with advanced NSCLC (NCT02099058), forty c‐Met–positive patients (33 nonsquamous, six squamous, one mixed histology) were included in the final analysis.^193^ Nine (23%) of these patients experienced objective responses with a mPFS of 5.2 months and a median response duration of 8.7 months. On January 4, 2022, the US FDA granted breakthrough therapy designation to Teliso‐V for patients with advanced/metastatic EGFR wild‐type nonsquamous NSCLC who have disease progression during or after platinum‐based therapy and c‐Met overexpression, based on supporting data from the ongoing phase II LUMINOSITY clinical trial (NCT03539536) presented at AACR in 2021. In the nonsquamous EGFR wild‐type subgroup of this trial (*n* = 37), the ORR assessed by an independent center was 35.1%. The ORR of the c‐Met overexpression subgroup (*n* = 13) was 53.8%, while that of the moderate c‐Met subgroup was 25.0%. The DoR in this cohort was 6.9 months. The EGFR mutation subgroup (*n* = 30) had an ORR of 13.3% and showed remission only in patients with high c‐Met (*n* = 22). However, in the squamous cell subgroup (*n* = 21), the ORR was 14.3% with a DoR of 4.4 months. All responses observed were PRs.

In the Lung‐MAP S1400K study (NCT03574753), Teliso‐V did not meet the expected response rate endpoint, achieving only 9% in patients with c‐Met‐positive squamous cell carcinoma.^194^ However, in patients with dual c‐Met/EGFR mutations, Teliso‐V in combination with other TKIs (erlotinib) demonstrated promising antitumor activity and acceptable toxicity levels (NCT02099058). The mPFS was 5.9 months (95% CI, 2.8–not reached) with an ORR of 32.1%. Among EGFR‐mutated patients, those with c‐Met overexpression (*n* = 15) had an ORR of 52.6%.^195^ This treatment approach is also under investigation in combination with osimertinib in the EGFR‐positive cohort (NCT06093503).

### 3.6 Nectin‐4

Nectin‐4, a protein encoded by the poliovirus receptor‐related‐4 gene, is a member of the Nectins family (Nectins 1−4). It belongs to the Ca^2+^‐independent immunoglobulin‐like protein group and plays a crucial role in the formation and maintenance of cell‐cell adherence and tight junctions through homophilic/heterophilic interactions.^196^, ^197^, ^198^ While other Nectins are predominantly expressed in adult healthy tissues, Nectin‐4 is specifically enriched in normal embryonic and fetal tissues.^196^, ^199^ Notably, recent studies have shown that Nectin‐4 is overexpressed in various malignant tumors, contributing to disease progression and poor prognosis in cancers such as urothelial cancer, breast cancer, pancreatic cancer, TNBC, and bladder cancer.^200^, ^201^, ^202^, ^203^, ^204^, ^205^, ^206^, ^207^, ^208^ The overexpression of Nectin‐4 has been linked to promoting tumor cell proliferation, differentiation, angiogenesis, migration, invasion, epithelial to mesenchymal transition, and DNA repair by activating the PI3K/Akt pathway.^209^, ^210^, ^211^

Enfortumab vedotin, also known as ASG‐22CE, is a new type of fully humanized monoclonal ADC that is linked to the microtubule‐disrupting agent MMAE through a protease‐cleavable linker.^212^ This drug is designed to specifically target cancer cells that express Nectin‐4, leading to internalization of the ADC–Nectin‐4 complex and subsequent cleavage of MMAE. This process ultimately disrupts the microtubule network within the cells, triggering apoptosis.^213^

In the assessment of tolerability and antitumor activity, 201 heavily pretreated patients with Nectin‐4–expressing solid tumors (primary tumors of bladder, renal, pelvis, ureter, lung, ovary, colon, and appendix) were administered enfortumab vedotin in the EV‐101 clinical trial (NCT02091999).^214^ Among the 112 patients with metastatic urothelial carcinoma, the ORR was 43%, and the DoR was 7.4 months. The median OS was 12.3 months with a 1‐year OS rate of 51.8%. In December 2019, the US FDA granted accelerated approval to enfortumab vedotin for patients with locally advanced or metastatic urothelial carcinoma that had progressed despite treatment with two previous therapies based on findings from the EV‐201 clinical trial (NCT03219333).^215^, ^216^ Patients in this cohort had initially received platinum‐based doublet chemotherapy as the first‐line treatment, followed by PD‐1/PD‐L1 inhibitors. Subsequently, enfortumab vedotin, instead of taxane agents like paclitaxel and docetaxel, was administered to all 125 participants as the third‐line therapy. Treatment with enfortumab vedotin effectively inhibited tumor growth in most patients, resulting in an ORR of 44% (55 out of 125, 95%CI, 35.1–53.2), a CR rate of 12% (15 out of 125), and a median DoR of 7.6 months (range: 0.95–11.3+).

In the cohort 2 of the EV‐201 clinical trial (NCT03219333), researchers evaluated the efficacy and safety of enfortumab vedotin in cisplatin‐ineligible patients with urothelial carcinoma in the post‐immunotherapy setting. The primary endpoint was the ORR, and the majority of participants were elderly individuals with renal impairment. Results from Cohort 2 revealed that among patients treated with the ADC, the confirmed ORR was 52%, with a CR rate of 20%, and a median DoR of 10.9 months. The mPFS and median OS were 5.8 and 14.7 months, respectively.^216^ This study presents potentially more effective and promising treatment options for a patient population with significant unmet needs. Various clinical trials investigating enfortumab vedotin in the treatment of urothelial cancer have gained momentum following the launch of EV‐201. In a phase III trial (EV‐301) of enfortumab vedotin in previously treated advanced urothelial carcinoma, 307 participants were assigned to receive chemotherapy and 301 to receive enfortumab vedotin (NCT03474107).^217^ Median OS was prolonged with enfortumab vedotin compared with chemotherapy (12.88 vs. 8.97 months; HR = 0.70, *p* = 0.00142). Median PFS in the enfortumab vedotin group was also longer than that with chemotherapy (5.55 vs. 3.71 months; HR = 0.62, *p* < 0.00001). Both groups exhibited similar rates of treatment emergent adverse events and grade ≥3 events. Even after a median follow‐up of 2 years, the benefits in terms of PFS, OS, and overall response remained consistent.^218^

In the EV‐302 clinical trials (NCT04223856), the combination treatment of enfortumab vedotin and pembrolizumab showed significantly better outcomes compared with chemotherapy for patients with untreated locally advanced or metastatic urothelial carcinoma.^219^ The median PFS and OS were longer in the enfortumab vedotin—pembrolizumab group than in the chemotherapy group (PFS: 12.5 vs. 6.3 months; HR = 0.45; 95% CI, 0.38–0.54; *p* < 0.001; OS: 31.5 vs. 16.1 months; HR = 0.47; 95% CI, 0.38–0.58; *p* < 0.001, respectively).

### 3.7 CEACAM5

CEACAM5, also known as CEA or CD66e, is a member of the immunoglobulin supergene family of adhesion molecules.^220^ It consists of a single N domain, six immunoglobulin constant‐like domains, and a glycosyl phosphatidylinositol anchor, allowing it to be located on the cell membrane and participate in intercellular adhesion and signaling.^221^, ^222^, ^223^ While CEACAM5 shows limited expression in normal adult tissues, it is highly expressed in various cancers of the gastrointestinal tract, breast, pancreas, genitourinary system, and respiratory system.^224^, ^225^, ^226^, ^227^ This makes CEACAM5 a valuable prognostic marker and potential therapeutic target for CEA‐positive cancers.^228^

Tusamitamab ravtansine (SAR408701) is a humanized antibody targeted towards CEACAM5. It is composed of the humanized monoclonal antibody (SAR408377) covalently linked to a cytotoxic maytansinoid DM4 payload, which is a microtubule‐destabilizing agent. This linkage is achieved through a cleavable N‐succinimidyl 4‐(2‐pyridyldithio) butanoate linker.^229^, ^230^ The DAR of tusamitamab ravtansine is 3.8.

The first‐in‐human dose‐escalation study of tusamitamab ravtansine included 31 patients with locally advanced or metastatic solid tumors (NCT02187848).^230^ Out of all dose levels and cancer types, 29 participants were evaluated for tumor response, revealing 3 (9.7%) PR, 11 (35.5%) SD, and 13 (41.9%) PD. Additionally, two patients did not exhibit CR or disease progression. Among the two colorectal cancer patients who showed PRs, one had a KRASG12V mutation and both had 2+ membrane CEACAM5 expression in 100% of cancer cells. The third patient with a PR had stomach cancer but lacked CEACAM5 expression in a limited amount of tumor tissue. In the CARMEN‐LC03 clinical trial (NCT04154956), tusamitamab ravtansine did not achieve the dual primary endpoint of PFS and OS as a monotherapy compared with docetaxel, as determined by the Independent Data Monitoring Committee. As a result, subsequent clinical trials involving tusamitamab ravtansine have been halted as of December 2023.

### 3.8 TF

TF, also known as coagulation factor III or CD142, is a transmembrane glycoprotein that plays a crucial role in initiating exogenous clotting pathways. Comprising 263 amino acid residues across extracellular, transmembrane, and intracellular regions,^231^, ^232^ TF kickstart the blood clotting cascade by binding to factor VII/VIIa.^232^ Recent studies have shown that the interaction between TF and VIIa (FVIIa) can impact angiogenesis, cancer stem cell activity, tumor growth, invasion, and metastasis through signal transduction pathways.^233^, ^234^, ^235^, ^236^, ^237^, ^238^, ^239^, ^240^

Tisotumab vedotin, a fully human TF‐specific monoclonal antibody linked to the cytotoxic MMAE payload, demonstrates promising antitumor activity.^241^ Upon binding to TF, the antibody is internalized into tumor cells, where it is cleaved by lysosomal proteases, releasing MMAE and ultimately inducing cell division inhibition and apoptosis. In the InnovaTV 201 clinical trial (NCT02001623), 27 patients with relapsed, advanced, or metastatic solid tumors underwent dose‐escalation of tisotumab vedotin, followed by 147 patients receiving a dose of 2.0 mg/kg in the dose‐expansion phase.^242^ The confirmed ORR in the dose‐expansion phase was 15.6%, with all responses being partial. The median DoR was 5.7 months (95% CI, 3.0–9.5) and the mPFS was 3.0 months (95% CI, 2.8–4.1). In the InnovaTV 204/GOG‐3023/ENGOT‐cx6 trials involving 101 patients with recurrent or metastatic cervical cancer, tisotumab vedotin showed an ORR, with 24 patients achieving a confirmed response, including 7 CRs and 17 PRs. The most common treatment‐related adverse events included alopecia (38%), epistaxis (30%), nausea (27%), fatigue (26%), and conjunctivitis (26%). Four deaths were reported, one of which was attributed to septic shock associated with the drug. Following the positive outcomes of the InnovaTV 201 and InnovaTV 204 trials, tisotumab vedotin received accelerated approval from the US FDA for patients with recurrent or metastatic cervical cancer.^243^ Results from the innovaTV 205/GOG‐3024/ENGOT‐cx8 study (NCT03786081) showed that tisotumab vedotin in combination with bevacizumab, carboplatin, or pembrolizumab had tolerable safety profiles and promising antitumor effects in both treatment‐naive and pretreated recurrent or metastatic cervical cancer patients. In the group treated with tisotumab vedotin plus carboplatin as first‐line therapy (*n* = 32), the ORR was 56.3%, the DCR was 93.8%, and the clinical benefit rate was 81.3%. The median DoR was 8.6 months (95% CI, 4.2–11.5). The mPFS was 6.9 months (95% CI, 4.0–11.1) and mOS was not reached at data cutoff (death rate: 42.4%). Among treatment‐naive patients who received tisotumab vedotin and pembrolizumab (*n* = 31), the ORR was 41.9%, the DCR was 83.9%, and the clinical benefit rate was 74.2%. Median DoR and OS were not reached, while median PFS was 5.3 months (95% CI, 4.0–12.2). For patients who underwent 2nd or 3rd line therapy with tisotumab vedotin + pembrolizumab (*n* = 32), the ORR was 35.3%, the DCR was 73.5%, and the clinical benefit rate was 47.1%. The median DoR was 14.1 months (95% CI, 4.2–not reached). Median PFS and OS were 5.6 months (95% CI, 2.7–14.2) and 15.3 months (95% CI, 9.9–not reached; 21 deaths), respectively.

### 3.9 FRα

FRα), also known as FOLR1 or folate‐binding protein, is a member of the folate receptor family that binds to folate with high affinity and can transport folic acid through receptor‐mediated endocytosis.^244^, ^245^ FRα is typically low in normal tissues but highly expressed in various solid tumors, such as ovarian cancer (76–89%), TNBC (35–68%), endometrial cancer (20–50%), and lung cancer (75–90%).^246^ Research indicates that the FRα‐mediated signaling pathway plays a crucial role in tumorigenesis, involving processes like DNA repair, DNA synthesis, cell proliferation, and intracellular signaling. Inhibiting FRα may directly impede tumor growth,^247^, ^248^, ^249^ making it a promising target for tumor diagnosis and treatment.^246^, ^250^

MORAb‐202 is the first ADC designed to target FRα with a DAR of 4.0. This compound is created by linking an anti‐FRα monoclonal antibody to eribulin through an enzyme‐cleavable linker.^251^ Eribulin, known as a microtubule inhibitor, disrupts microtubule homeostasis, ultimately inhibiting cell division and effectively contributing to the destruction of cancer cells.

In the initial phase I trial of MORAb‐202 in patients with advanced solid tumors that are positive for FRα (NCT03386942), 45% of patients experienced treatment‐related adverse events of leukopenia and neutropenia.^252^ Additionally, one patient (0.9 mg/kg) experienced two grade 3 events of elevated serum alanine aminotransferase and γ‐glutamyl transferase levels. Out of the 22 patients enrolled, one patient with ovarian cancer achieved CR, nine patients (41%) had PR, eight patients (36%) had SD, and the remaining four patients (18%) showed disease progression. MORAb‐202 also demonstrated significant inhibition of the growth of FRα‐expressing breast cancer cell lines.^253^ These promising results with MORAb‐202 in FRα‐positive solid tumors have led to further investigations into its clinical utility.

Mirvetuximab soravtansine (IMGN853) is an ADC primarily used in the treatment of certain types of cancer, especially in ovarian cancer patients with high FRα expression.^254^ The drug is composed of a FRα‐binding antibody (mirvetuximab), a lysable linker, and the maytansinoid payload DM4 (soravtansine). Mirvetuximab soravtansine binds selectively to FRα with high affinity and is then internalized through antigen‐mediated endocytosis. Once inside FRa‐expressing tumor cells, DM4 is released through proteolytic cleavage. DM4 disrupts the intracellular microtubule network, causing cell cycle arrest and apoptosis.^255^ Due to its electroneutral and lipophilic characteristics, DM4 can diffuse across cell membranes and induce cell death in neighboring antigen‐negative cells, a phenomenon known as the “bystander effect.” This property is crucial for mirvetuximab soravtansine as it enables a cytotoxic effect even in cells lacking FRα expression on their surface.^256^

This phase I expansion cohort study examined the safety and clinical effectiveness of mirvetuximab soravtansine in forty‐six heavily pre‐treated patients with FRα‐positive and platinum‐resistant ovarian cancer (NCT01609556).^257^ The most common adverse events were diarrhea (44%), blurred vision (41%), nausea (37%), and fatigue (30%), all of which were generally mild (≤grade 2). Grade 3 hypotension and fatigue were reported in two patients each (4%). The confirmed ORR was 26%, with one CR and 11 PRs. The mPFS was 4.8 months (95% CI, 3.9–5.7) regardless of FRα expression. Interestingly, patients with <4 prior lines of therapy had a mPFS of 6.7 months (95% CI, 3.9–8.7) and an ORR of 39%. Following the promising preliminary data, an open‐label randomized controlled phase III trial (FORWARD I) was initiated (NCT02631876).^258^ This trial enrolled patients (*n* = 366) with <4 prior lines of therapy and FRα‐positive tumors (medium and high expression). Patients were randomly assigned to receive mirvetuximab soravtansine at 6 mg/kg or the physician's choice of treatment (including paclitaxel, pegylated liposomal doxorubicin, or topotecan). While all secondary endpoints, such as ORR (22 vs. 12%), CA125 response (51 vs. 27%), post‐PFS (median 10.0 vs. 8.4 months), and quality of life (32 vs. 14%), showed significant improvements, the primary endpoint of PFS did not meet expectations.^258^

While the results from FORWARD I were disappointing, the HRs for PFS and OS indicated potential benefits of mirvetuximab soravtansine in the high FRα subgroup, although without statistical significance.^258^ Two additional studies were conducted focusing on mirvetuximab soravtansine in platinum‐resistant cancer patients with high FRα levels. The SORAYA study (NCT04296890), which exclusively enrolled FRα‐high patients, consisted of 106 individuals with high‐grade serous ovarian cancer, primary peritoneal cancer, or fallopian tube cancer. Among these patients, 51% had undergone three prior lines of therapy, and 48% had received a prior poly ADP‐ribose polymerase inhibitor.^254^ All participants had previously received bevacizumab and subsequently received single‐agent mirvetuximab soravtansine at a dose of 6 mg/kg. Five patients achieved CRs, and 29 achieved PRs, resulting in a confirmed ORR of 32.4% and a DCR of 51.4%. The majority of patients (71.4%) experienced tumor reduction. The mPFS was 5.5 months (95% CI, 3.8–6.9), and the median OS was 13.8 months (95% CI, 12.0–not reached). Subsequently, mirvetuximab soravtansine was approved by the US FDA for FRα‐positive platinum‐resistant epithelial ovarian, fallopian tube, or peritoneal cancer patients who had received 1–3 prior systemic therapies.^254^

In the phase III MIRASOL trial (NCT04209855), researchers compared the efficacy of mirvetuximab soravtansine versus chemotherapy in patients with platinum‐resistant, advanced high‐grade epithelial ovarian, primary peritoneal, or fallopian tube cancers expressing high FRα.^259^ Out of the 453 patients enrolled, 14% had one prior line of therapy, 39% had two prior lines, and 47% had three prior lines. A majority of patients had received prior treatment with bevacizumab (62%) and PARP inhibitors (55%). The primary endpoint of mPFS was 5.62 months (95% CI, 4.34–5.95) in the mirvetuximab soravtansine group compared with 3.98 months (95% CI, 2.86–4.47) in the chemotherapy group, with ORR of 42 and 16%, respectively (both *p *< 0.001). The mOS was longer in the mirvetuximab soravtansine group at 16.4 months compared with 12.7 months in the chemotherapy group (HR, 0.67, *p* = 0.0046).

In the recent multicohort FORWARD II (NCT02606305) study, data indicated that the ORR of mirvetuximab soravtansine combined with bevacizumab in the platinum‐sensitive recurrent ovarian cancer subgroup was 44%, with 5 CRs and 36 PRs.^260^, ^261^ The median DoR and PFS were 9.7 months (95% CI, 6.9–14.1) and 8.2 months (95% CI, 6.8–10.0), respectively. Encouraging results were seen across patients regardless of FRα expression levels or prior bevacizumab treatment.

In the context of platinum‐sensitive ovarian cancer, several recent clinical trials (including the PICCOLO trial, NCT05041257; MIROVA trial, NCT04274426; Study‐420, NCT04606914; and GLORIOSA trial, NCT05445778) have investigated the efficacy of mirvetuximab soravtansine.^262^ The results from the FORWARD II trial (NCT02606305) indicated that eighteen women with relapsed FRα‐positive and platinum‐sensitive ovarian cancer were treated with carboplatin in combination with mirvetuximab soravtansine.^263^ Following carboplatin discontinuation, thirteen patients continued with mirvetuximab soravtansine maintenance, resulting in three complete and nine PRs. The ORR was 71%, with a mPFS of 15 months.

### 3.10 Delta‐like protein 3 (DLL3)

DLL3 is a single transmembrane protein that is anchored to the cell surface and belongs to the Notch ligand family. The human DLL3 protein consists of 619 amino acids and its complete structure includes a DSL domain, an intracellular domain, and six EGF‐like repeat domains. This ligand, DLL3, exerts its biological functions either directly or indirectly by binding to the Notch receptor. Unlike other Notch ligands, DLL3 has an inhibitory effect on the Notch pathway.^264^ Through interactions with Notch receptors, DLL3 inhibits the activation of the Notch signaling pathway. It can also bind to DLL1 and Notch1 receptors to inhibit modification and promote the degradation of Notch1 receptor, thus inhibiting the activation and upregulation of the Notch signaling pathway.^265^, ^266^ Research has shown that DLL3 is primarily expressed in neuroendocrine tumors, such as SCLC, large cell neuroendocrine carcinoma (LCNEC), gastrointestinal neuroendocrine tumor, small cell bladder cancer, glioblastoma multiforme, metastatic castrated prostate cancer, lung neuroendocrine tumor, among others.^267^, ^268^, ^269^, ^270^, ^271^ Particularly, more than 80% of SCLC cases exhibit positive expression of DLL3, leading to enhanced migration and invasion of SCLC cells.^272^ However, DLL3 is rarely expressed in normal healthy tissues, making it a promising target for lung cancer therapies.^273^

Rovalpituzumab tesirine (Rova‐T, S16LD6.5) is a novel ADC that targets DLL3, a protein found in certain tumor cells. This ADC is made up of a humanized IgG1 monoclonal antibody, a cytotoxic drug called pyrrole benzodiazepine (PBD) (D6.5), and a protease‐cleavable connector.^274^, ^275^ The antibody component specifically recognizes DLL3, allowing the chemotherapy drug to be delivered directly into the cell, causing DNA damage and inhibiting tumor cell growth.^275^ In a phase I study (NCT01901653) involving patients with SCLC and LCNEC, Rova‐T showed promising antitumor activity with manageable side effects.^276^ Among the patients who received Rova‐T, the ORR was 18% and the DCR was 68%. Notably, patients with positive DLL3 had a higher response rate, with an ORR of 38% for those who received the optimal dose; among patients who received the optimal dose and were positive for DLL3, the ORR was as high as 55%, and the DCR was as high as 91%.^276^ However, in a subsequent phase II TRINITY study for SCLC patients with multiple prior treatments (NCT02674568), the ORR was lower at 12.4% in all participants, 14.3% with ≥25% of tumor cells positive for DLL3, and 13.2% with ≥75% of tumor cells positive for DLL3, respectively.^277^ The initial phase I study did not yield the desired effect for Rova‐T. The phase II MERU Study aimed to evaluate Rova‐T's efficacy as maintenance therapy for patients with extensive‐stage–SCLC following platinum‐based chemotherapy. While patients receiving Rova‐T had a longer mPFS compared with those on placebo (4.0 vs. 1.4 months, HR = 0.48, *p* < 0.001), there was no significant difference in mOS between the Rova‐T and placebo groups (8.5 vs. 9.8 months, HR = 1.07, *p* = 0.537).

The clinical trial did not achieve its primary endpoint and was stopped prematurely. Results from the phase III TAHOE study indicated that Rova‐T had a lower OS but higher incidence of side effects (such as serosal effusions, photosensitivity reaction, and peripheral edema) compared with topotecan as a second‐line therapy in patients with DLL3‐high SCLC (NCT03061812).^278^ The median OS (primary endpoint) and PFS were 6.3 months (95% CI, 5.6–7.3) and 3.0 months (95% CI, 2.9–3.6) in the Rova‐T group, and 8.6 months (95% CI, 7.7–10.1) and 4.3 months (95% CI, 3.8–5.4) in the topotecan group, respectively. Additionally, the ORR was only 15% (42 out of 287) in the Rova‐T group, compared with 21% (27 out of 129) in the topotecan group.^278^

### 3.11 B7‐H3

B7‐H3, also known as CD276 or B7RP‐2, is a transmembrane glycoprotein with type I structure, consisting of extracellular, transmembrane, and short intracellular domains.^279^, ^280^ Initially identified for its ability to stimulate T cell responses and IFN‐γ production, recent studies suggest that B7‐H3 predominantly acts as an immune cosuppressor, aiding tumor cells in evading immune surveillance.^279^, ^281^ It is prominently expressed in various solid tumors, while being minimally present in normal tissues. Consequently, elevated levels of B7‐H3 are correlated with a poorer prognosis in cancer patients.^281^, ^282^

HS‐20093 is an ADC specifically targeting tumors with high B7‐H3 expression. It consists of a fully human anti‐B7‐H3 monoclonal antibody of the IgG1 subtype linked to a small molecule toxin topoisomerase I inhibitor through a protease‐cleavable linker.^283^, ^284^ In a study involving 40 patients with NSCLC, SCLC, sarcoma, and other advanced solid tumors who were treated with varying doses of HS‐20093, preliminary efficacy results showed 14 PRs, with nine confirmed PRs and five additional PRs pending confirmation irrespective of B7‐H3 levels (NCT05276609). Furthermore, SD was observed in 19 additional patients. Treatment‐emerged adverse events (TEAEs) were reported in all enrolled patients, with the most common TEAEs occurring in over 30% of patients including leukopenia, neutropenia, anemia, pyrexia, nausea, thrombocytopenia, hypoalbuminemia, vomiting, lymphopenia, infusion‐related reactions, and fatigue. Among a subset of nine SCLC patients, seven PRs were observed, resulting in a response rate of 77.8% with a median depth of response of 50.5%.^283^ These findings suggest that HS‐20093 could be a promising candidate drug for patients with B7‐H3‐expressing solid tumors.

In a phase I dose‐escalation study (NCT04145622), ifinatamab deruxtecan (formerly known as DS‐7300a) showed promising results in 127 patients with metastatic castration‐resistant prostate cancer (mCRPC), esophageal squamous cell carcinoma, squamous cell lung cancer, and SCLC.^285^ Adverse events were reported in 98% of patients, with nausea (61%), infusion‐related reactions (35%), and vomiting (31%) being the most common. Notably, one patient experienced a grade 5 interstitial lung disease at a dose of 16 mg/kg. The recommended dose of DS‐7300a was 12 mg/kg every 3 weeks, with an ORR of 33% and a DCR of 71.4% across all tumor types. Specifically, responses were observed in 78% (seven out of nine) of SCLC cases, 40% (two out of five) of squamous cell NSCLC cases, and 38% (16 out of 42) of mCRPC cases.^286^ The most common adverse events reported were nausea (65.5%), infusion reactions (34.5%), and fatigue (34.5%).^284^

Vobramitamab duocarmazine, formerly known as MGC018, is an ADC that targets B7‐H3. It consists of valine–citrulline–seco duocarmycin hydroxybenzamide azaindole as the cleavable linker‐duocarmycin payload, conjugated to an anti‐B7‐H3 humanized monoclonal antibody through reduced interchain disulfides, with an average DAR of 2.7.^287^ In a phase I dose escalation study (NCT03729596) involving 29 patients with various tumor types, all participants experienced adverse events, with common ones being anemia, neutropenia, fatigue, hyperpigmentation, infusion‐related reactions, nausea, and palmar plantar erythrodysesthesia.^288^ Among three melanoma patients, some showed a 20% or greater reduction in target lesion size, although without achieving PRs. In another phase I study (NCT03761017), four patients with measurable mCRPC experienced reductions in target lesions, and half of the patients had a more than 50% reduction in prostate‐specific antigen levels.^289^ One melanoma patient achieved a PR. Most patients (43 out of 49) experienced adverse events. The results of vobramitamab duocarmazine so far have shown mild toxicities and initial signs of clinical antitumor activity in solid tumors.

### 3.12 CD19

B lymphocyte antigen CD19, also known as B lymphocyte surface antigen B4, T cell surface antigen Leu‐12 and CVID3, is a transmembrane protein encoded by the CD19 gene. In humans, CD19 is expressed in all B lineage cells except plasma cells and follicular dendritic cells. CD19 plays dual roles in human B cells, serving as an adaptor protein to recruit cytoplasmic signaling proteins to the membrane and working within the CD19/CD21 complex to lower the threshold of the B‐cell receptor signaling pathway.^290^ Due to its presence in all B cells, CD19 serves as a biomarker for B lymphocyte development, lymphoma diagnosis, and can be targeted therapeutically for hematological malignancies. Loncastuximab tesirine (ADCT‐402) is a CD19‐directed ADC consisting of an anti‐CD19 monoclonal antibody randomly linked to a PBD dimer cytotoxic alkylating agent (SG3199) through a cathepsin‐cleavable valine‐alanine linker.^291^ Once internalized, the PBD binds irreversibly to CD19, releasing conjugates within tumor cells. These conjugates bind to DNA, cross‐linking the DNA double strands, inhibiting DNA strand separation, and ultimately leading to tumor cell death.

The safety and initial antitumor efficacy of loncastuximab tesirine were first documented in 2019 among patients with relapsed or refractory B‐cell non‐Hodgkin lymphoma (NCT02669017).^292^, ^293^ Nearly all patients (98.9%) experienced treatment‐related adverse events, with the most common being hematologic abnormalities (such as decreased platelet and neutrophil counts), fatigue, edema, liver test abnormalities, nausea, rash, and dyspnea (reported in ≥20% of patients). Among evaluable patients, the ORR was 59.4% at doses ≥120 µg/kg, with a CR rate of 40.6% and a PR rate of 18.8%. The median DoR, PFS, and OS at all doses were 4.8, 5.5, and 11.6 months, respectively. In the pivotal phase II LOTIS‐2 study (NCT03589469), heavily pretreated patients with relapsed or refractory diffuse large B‐cell lymphoma (DLBCL) treated with loncastuximab tesirine had a mOS of 9.5 months and a mPFS of 4.9 months at the time of data cutoff.^294^, ^295^ 48.3% of the 145 patients achieved an objective response, with 24.8% achieving CRs. Patients who achieved CR had not reached median PFS or OS, with 24‐month OS and PFS rates of 68.2 and 72.5%, respectively. The US FDA has expedited the approval of loncastuximab tesirine for the management of adult patients with relapsed or refractory large B‐cell lymphoma who have undergone two or more lines of systemic treatments, including those with other diffuse tumors of unknown origin, large B‐cell lymphoma, large B‐cell lymphoma originating from low‐grade lymphoma, and high‐grade lymphoma.^296^

### 3.13 CD22

CD22, a type I transmembrane glycoprotein and member of the sialic acid‐binding immunoglobulin‐like lectin family,^297^ acts as an inhibitory coreceptor of the B cell receptor (BCR) to negatively regulate B cell activation signals.^298^ Specifically binding to glycoprotein ligands containing α‐2,6‐linked sialic acid, CD22 can activate BCR, phosphorylate tyrosine in the immunoreceptor tyrosine inhibitory motif within its cytoplasmic region, trigger downstream signaling molecules, inhibit calcium ion influx, and ultimately weaken BCR signals.^299^, ^300^, ^301^ Additionally, CD22 plays a role in the homing process of B cells.^302^ Due to its relatively specific expression on the surface of B cells, CD22 has emerged as a promising target for regulating B cell immunity and treating certain B cell tumors.^303^

Inotuzumab ozogamicin (InO) is a human CD22‐directed ADC consisting of a humanized anti‐CD22 antibody linked to the potent cytotoxic agent calicheamicin.^304^ InO can bind to CD22‐expressing tumor cells, leading to internalization of the ADC‐CD22 complex. Subsequently, hydrolysis of the covalent bond of N‐acetyl‐γ‐kazimycin diformylhydrazide occurs, inducing double‐strand DNA breakage and cell death.^305^, ^306^ Initial studies in 2010 showed promising antitumor activities of InO in CD22‐positive B‐cell non‐Hodgkin's lymphoma, with the main toxicity being thrombocytopenia (NCT00073749). The mPFS was 317 days for follicular non‐Hodgkin's lymphoma patients and 49 days for diffuse large B‐cell lymphoma patients.^307^, ^308^ Final results from the randomized phase III INO‐VATE study (NCT01564784) revealed a significantly higher CR or CR with incomplete hematologic recovery (CRi) rate with InO compared with standard‐of‐care chemotherapy (73.8 vs. 30.9%; *p* < 0.0001).^309^, ^310^, ^311^, ^312^ The median OS was 7.7 months with InO and 6.2 months with chemotherapy, with 2‐year OS rates of 22.8 and 10.0%, respectively (HR, 0.75; 97.5% CI, 0.57–0.99; *p* = 0.0105). A higher percentage of patients treated with InO proceeded directly to hematopoietic stem cell transplantation after achieving CR/CRi compared with those receiving chemotherapy (39.6 vs. 10.5%; *p* < 0.0001). The most common adverse events in both groups were hematologic. In addition to monotherapy, combination treatments of InO with chemotherapy have shown promise in patients with acute lymphocytic leukemia or B‐cell lymphoma. Combining InO with low‐intensity chemotherapy, with or without blinatumomab, demonstrated positive effects on PFS in older patients with B‐cell acute lymphocytic leukemia (NCT01371630).^313^ The 2‐year PFS and 5‐year PFS rates were 58.2% (95% CI, 46.7–68.2) and 44.0% (31.2–54.3), respectively in older patients with newly diagnosed Philadelphia chromosome‐negative B‐cell acute lymphocytic leukemia.^313^ The most common grade 3−4 adverse events were thrombocytopenia (78%) and febrile neutropenia (32%). A total of 46 patients (78%) responded to treatment, with 35 of them (59%) achieving CRs. The overall minimal residual disease negativity rate among responders was 82%.^314^ However, a study comparing InO plus rituximab to rituximab plus bendamustine or gemcitabine in patients with relapsed/refractory B‐cell non‐Hodgkin lymphoma showed no superiority of InO plus rituximab based on the results of a, open‐label, phase III study (NCT01232556).^315^ Similarly, the combination of InO and rituximab as a salvage regimen for transplant‐eligible patients with relapsed/refractory diffuse large B‐cell lymphoma yielded lower than expected favorable outcomes.^316^ The 1‐year and 2‐year PFS rates for all enrolled patients were only 28.9 and 25.3%, respectively, with a median PFS of 3.0 months.

### 3.14 CD30

CD30 (TNFRSF8) is a membrane protein closely associated with cell proliferation and death.^317^ When CD30 molecules are stimulated, they undergo receptor trimerization and signal transduction, activating the nuclear factor‐κB (NF‐κB) pathway. Additionally, CD30 is involved in the MAPK pathway, specifically ERK1 and ERK2, which promote antiapoptotic and pro‐survival effects in tumor cells.^318^ Furthermore, there appears to be a positive feedback loop between the MAPK/ERK pathway and NF‐κB, which not only contributes to cell survival but also upregulates CD30 expression.^319^, ^320^ There seems to be a positive feedback loop between the MAPK/ERK pathway and NF‐κB, enhancing cell survival and increasing CD30 expression. This indicates that CD30 expression in tumor cells may promote proliferation and inhibit cell death. In healthy cells, CD30 is typically not expressed or is expressed at low levels on activated T cells and B cells. Conversely, CD30 is often highly expressed on the surface of Hodgkin lymphoma and anaplastic large cell lymphoma cells.^321^, ^322^, ^323^, ^324^ This expression pattern aligns with the ideal target antigen characteristics, showing high consistency on target cells and low expression in normal tissues.^325^ Such distinct expression patterns can help minimize off‐target toxicity in nontumor tissues.

The ADC of brentuximab vedotin is composed of an anti‐CD30 monoclonal antibody, a protease‐cleavable linker, and MMAE, with a DAR of 4.^326^ Brentuximab demonstrates stability in the bloodstream. Upon binding to the CD30 receptor on cancer cells, it is endocytosed, releasing MMAE within the cancer cells. MMAE disrupts the microtubule network in cancer cells, halting the mitotic cycle and triggering cell apoptosis.^327^ Furthermore, MMAE can also enter neighboring cancer cells through a bystander effect, contributing to the elimination of other cancer cells.^328^ The US FDA has granted accelerated approval for the use of this CD30 monoclonal antibody in treating relapsed or refractory Hodgkin's lymphoma and anaplastic large cell lymphoma.^329^

Brentuximab vedotin demonstrated manageable toxic effects and elicited objective responses in a majority of patients with relapsed or refractory CD30‐positive lymphomas during the initial phase I trial (NCT00430846).^330^ Among the 44 evaluable patients, 17 showed objective responses, including 11 CRs, 6 PRs, and 19 SDs. The median PFS was estimated to be 5.9 months, with a DoR of 17.3 months for the 17 patients who exhibited objective responses (ranging from 0.6 to >19.5 months). The most common side effects, mostly grade 1 or 2, included fatigue (16, 36%), pyrexia (15, 33%), peripheral neuropathy, nausea, diarrhea, neutropenia, and peripheral neuropathy (10, 22% each). In the phase III ECHELON‐1 study (NCT01712490), treatment‐naive patients with stage III–IV classic Hodgkin lymphoma (cHL) received brentuximab vedotin in combination with doxorubicin, vinblastine, and dacarbazine (A+AVD) or bleomycin plus AVD (ABVD) as first‐line therapy.^331^ With a median follow‐up of 24.9 months, the primary endpoint of PFS was significantly higher in the A+AVD group compared with the ABVD group (HR, 0.77, *p* = 0.03). The 2‐year PFS rates were 82.1% (95% CI, 78.7–85.0%) and 77.2% (95% CI, 73.7–80.4%) in the respective groups. All secondary efficacy endpoints (OS, CR rate, ORR, and DoR rate) favored the A+AVD regimen. As a result, the combination of brentuximab vedotin with AVD was recommended as a first‐line treatment for cHL due to its superior efficacy and lower incidence of severe toxicity in the ECHELON‐1 study.^331^ Furthermore, the AETHERA clinical trial (NCT01100502) confirmed the role of brentuximab vedotin as a consolidative treatment option for adult cHL patients at high risk of relapse or progression after autologous hematopoietic stem‐cell transplantation.^332^, ^333^, ^334^ During the 5‐year follow‐up period, patients treated with brentuximab vedotin demonstrated a durable PFS benefit, with a 5‐year PFS rate of 59% (95% CI, 51–66) compared with 41% (95% CI, 33–49) in the placebo group (HR = 0.521). Furthermore, patients with at least two risk factors in the brentuximab vedotin group exhibited significantly higher PFS rates at 5 years when compared with patients in the control arm (HR, 0.424; 95% CI, 0.302–0.596).

The ECHELON‐2 (NCT01777152) study is the largest prospective, randomized, double‐blind trial comparing the effectiveness and safety of standard CHOP regimen with alternatives containing CD30‐targeted agents (brentuximab vedotin plus cyclophosphamide, doxorubicin, and prednisone, A+CHP) as initial treatment for sALCL and other CD30‐positive peripheral T‐cell lymphomas.^335^, ^336^ In the 5‐year update of ECHELON‐2, the 5‐year PFS and OS rates with A+CHP were 51.4% (95% CI, 42.8–59.4%) and 70.1% (95% CI, 63.3–75.9%), respectively, compared with 43.0% (95% CI, 35.8–50.0%) and 61.0% (95% CI, 54.0–67.3%) with CHOP. The safety profile was similar in both groups, with manageable hematologic toxicity being the primary concern. Peripheral neuropathy was reported in 67% of cases but was generally tolerable. The US FDA has approved the combination of brentuximab vedotin and CHP chemotherapy regimen (cyclophosphamide, doxorubicin, and prednisone) for the frontline treatment of sALCL or other CD30‐positive PTCL in adults.^337^

Adult patients with primary cutaneous anaplastic large cell lymphoma (C‐ALCL) or CD30‐expressing mycosis fungoides who had previously undergone systemic therapy were eligible to receive brentuximab vedotin based on the findings of the ALCANZA trial (NCT01578499).^338^, ^339^, ^340^, ^341^ The study demonstrated a significantly higher ORR lasting ≥4 months (ORR4; primary endpoint) with brentuximab vedotin compared with physician's choice (54.7 vs. 12.5%, *p* < 0.001). The median PFS in the brentuximab vedotin group was estimated to be 16.7 months, significantly longer than the physician's choice group (3.5 months, *p* < 0.001), with CR rates of 17.2 and 1.6%, respectively. Furthermore, 86% of patients experiencing any grade of peripheral neuropathy achieved complete remission (59.1%) or an improvement to grade 1 or 2 (27.3%) with brentuximab vedotin.

### 3.15 CD33

CD33 is a type I transmembrane glycoprotein with a molecular weight of 67 kDa that belongs to the Siglec family.^342^, ^343^ This molecule transmits inhibitory signals to the cell through its ITIM sequence, which blocks intracellular signaling pathways after cross‐linking or ligand binding.^344^, ^345^ CD33, known as a myeloid differentiation antigen, is predominantly found in myeloid blood cells, particularly in AML. Importantly, it is not expressed on the surface of normal hematopoietic stem cells and other mature cells, making CD33 a promising target for the treatment of myeloid leukemia.^346^, ^347^

GO, a recombinant humanized monoclonal antibody (hp67.6) linked to a potent cytotoxic antitumor antibiotic N‐acetylcalicheamicin (CLM), targets the antigen CD33.^348^, ^349^ The antibody binds specifically to the target antigen CD33, forming an antigen‐antibody complex that triggers endocytosis. This complex enters the cell via vesicles formed during endocytosis.^350^ The vesicles then fuse with intracellular lysosomes, allowing the antibodies to enter. In the acidic environment of the lysosomes, the linker connecting the antibody to the toxin breaks down, releasing CLM. CLM then enters the nucleus, causing the DNA double helix to break and inducing apoptosis in tumor cells.^351^ The US FDA approved GO in 2000 as the first ADC molecule targeting CD33 for treating patients with AML.^352^ A clinical response rate of 26% was observed in leukemias treated with GO.^353^ Administering GO to patients with CD33‐positive AML in first relapse led to complete remissions with a favorable safety profile.^4^, ^353^ The overall rate of remission was 30%, with 16% achieving complete remission and 13% achieving remission with incomplete platelet recovery.^4^

However, subsequent studies revealed that GO did not significantly enhance patient survival compared with traditional chemotherapy drugs and even led to higher rates of lethal hepatic toxicity. The phase III SWOG 106 study demonstrated a higher incidence of severe fatal liver injury in the GO treatment group (death rate, 5.7 vs. 1.4%) without showing a clear survival benefit.^354^, ^355^ These compelling results led to the early termination of the study, and the drug was subsequently withdrawn in 2010. Following dosage adjustments and extensive clinical trials, GO was reapproved by the US FDA in 2017 for newly diagnosed and relapsed refractory CD33‐positive AML patients.^356^, ^357^ In the phase III ALFA‐0701 trial (NCT 00927498), the addition of a reduced and fractionated dose of GO to standard chemotherapy regimens significantly improved event‐free survival (EFS) in adults with AML. The median EFS was 17.3 months (95%CI, 13.4–30.0) in the GO arm, compared with 9.5 months (8.1–12.0) in the standard 3+7 daunorubicin and cytarabine induction regimen (HR, 0.56, 95%CI, 0.42–0.76; *p* = 0.0002). This represented a 44% reduction in the risk of an event for patients in the GO group. Although a longer OS was observed in the GO arm, this difference was not statistically significant (27.5 vs. 21.8 months, *p* = 0.16). Importantly, it appeared that low CD33 expression (<30% of blasts positive) did not impact the EFS benefit with GO. The incidence of severe (grade ≥3) infection was similar in both treatment arms (77%). The number of patient deaths was also not significantly different between the GO arm (6; 4.6%) and the control arm (5; 3.6%). Furthermore, the addition of GO to chemotherapy demonstrated potential to improve EFS for children and adolescents with AML by reducing the risk of relapse, as evidenced by the phase III Children's Oncology Group trial AAML0531 (NCT00372593).^358^ The EFS was significantly enhanced with the addition of GO (3‐year EFS: 53.1 vs. 46.9%; HR: 0.83; 95% CI, 0.70–0.99; *p* = 0.04), although there was no significant OS benefit observed (3‐year rate: 69.4 vs. 65.4%; *p* = 0.39).^358^

### 3.16 CD79B

CD79B, along with CD79A, is crucial in initiating the signal transduction cascade activated by BCR.^359^, ^360^ This pivotal role leads to the internalization of the complex, subsequent transport to endosomes, and eventual antigen presentation.^361^ CD79B may enhance the phosphorylation of CD79A by recruiting kinases that phosphorylate CD79A or by conjugating proteins that bind to CD79A, thereby protecting it from dephosphorylation. Additionally, CD79B forms a heterodimer with the α chain to create a disulfide‐bonded complex.^362^ Furthermore, CD79B interacts with Lyn to further enhance its regulatory function in B cell signaling.^363^, ^364^, ^365^ As a signaling component of the B‐cell antigen receptor complex, CD79B is highly specific to the B‐cell lineage and is significantly expressed in various B‐cell lymphomas (including >95% DLBCL).^366^, ^367^ Preclinical studies have shown that targeting CD79B as a binding site is more effective than targeting CD79A.^368^ Consequently, CD79B has emerged as an ideal therapeutic target to harness the potential of ADC.^369^

Polatuzumab vedotin (Polivy) is a novel anti‐CD79B ADC that disrupts B cell division through a combination of a humanized anti‐CD79b monoclonal antibody, cytotoxic MMAE component, and a cleavable linker with a DAR of 3−4.^370^, ^371^ The antibody targets CD79B, which is overexpressed in DLBCL patients.^368^ Upon binding to CD79B, Polivy is internalized, leading to linker cleavage by lysosomal proteases and subsequent release of MMAE. MMAE then disrupts the microtubule network structure by binding to tubulin, ultimately inhibiting cell division and triggering apoptosis.^370^, ^372^ In June 2019, the US FDA granted accelerated approval to Polivy in combination with bendamustine plus rituximab for adults with DLBCL who have undergone second‐line or subsequent treatments.^370^

In a phase I study (NCT01290549), Polivy demonstrated acceptable safety and tolerability in patients with relapsed or refractory B‐cell non‐Hodgkin lymphoma but not in those with chronic lymphocytic leukemia.^373^ The accelerated approval of Polivy was based on an open‐label, multicenter, randomized phase II clinical study (GO29365, NCT02257567) that included 80 relapsed/refractory DLBCL patients who had received at least one prior treatment and were not suitable for autologous hematopoietic stem cell transplantation.^374^, ^375^ These patients were randomly assigned to receive standard treatment with bendamustine‐rituximab or bendamustine‐rituximab plus Polivy. The primary endpoint of CR was 17.5% in the standard therapy group and 40% in the Polivy group (*p* = 0.026). Moreover, the ORR in the Polivy arm was 63%, significantly higher than the 25% in the control group. Among patients in the Polivy group who achieved PRs or CRs, 64% experienced lasting responses for more than 6 months, with 48% maintaining remission for over 1 year. In contrast, these numbers were only 3 and 2% in the control group. Treatment with Polivy resulted in substantially higher response rates compared with standard therapy. Serious adverse reactions were reported in 60% of subjects, predominantly due to infections. The most common adverse reactions associated with Polivy included neutropenia, peripheral neuropathy, fatigue, thrombocytopenia, and pyrexia.

Moreover, the combination of Polivy plus R‐CHP has shown to reduce the risk of disease relapse, progression, or mortality in comparison with treatment with R‐CHOP among patients with previously untreated intermediate‐risk or high‐risk DLBCL, as demonstrated in the POLARIX trials (NCT03274492).^376^ The data analysis at the time of cutoff indicated a significantly lower risk of progression, relapse, or death in the pola‐R‐CHP group when compared with the R‐CHOP group (stratified HR, 0.73; 95% CI, 0.57–0.95; *p *= 0.02). The primary endpoint of the 2‐year PFS rate with Polivy plus R‐CHP was 76.7% (95% CI, 72.7–80.8), which was notably higher than the 70.2% (95% CI, 65.8–74.6) observed in the R‐CHOP group (HR, 0.73, *p *= 0.02). The OS at 2 years was comparable between the Polivy plus R‐CHP arm and the R‐CHOP arm (88.7 vs. 88.6%; HR, 0.94, *p* = 0.75). Notably, there were no significant differences in the safety profile between the two groups. Based on these findings, Polivy received US FDA approval for the treatment of DLBCL.^370^

## 4 ADC RESISTANCE MECHANISMS AND INTERVENTION STRATEGIES

**FIGURE 4:**
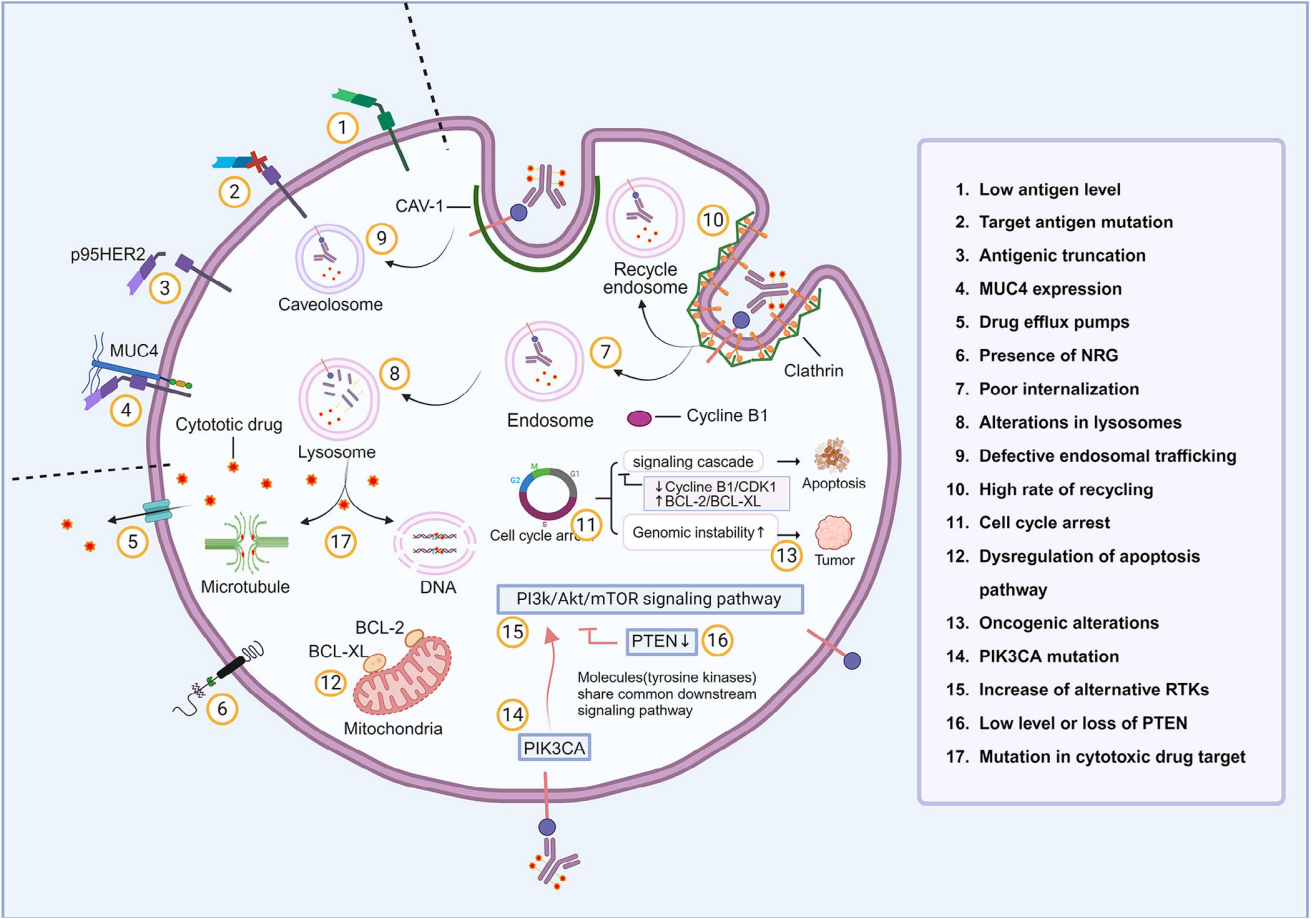
Mechanisms of ADCs resistance. The mechanisms of resistance to ADCs are numbered and illustrated. Detailed descriptions are presented in the text. ADCs, antibody–drug conjugates; NRG, Neuregulin‐1; RTKs, receptor tyrosine kinases; CAV‐1, caveolin‐1; HER2, human epidermal growth factor receptor 2; MUC4, Mucin 4; BCL‐2, B‐cell lymphoma‐2; BCL‐XL, B‐cell lymphoma‐XL; PIK3CA: the p110α catalytic subunit of phosphatidylinositol 3‐kinase (PI3K); PTEN, phosphatase and tensin homolog; PI3K/Akt/mTOR, phosphatidylinositol 3‐kinase/protein kinase‐B/mammalian target of the rapamycin.

Despite the promising advancements, the PFS of these ADCs varies from 2.0 to 28.8 months. Any abnormalities in the structure or selection of ADCs under therapeutic pressure can result in treatment resistance. The resistance mechanisms of ADCs may include: down‐regulation, deletion, or mutation of the target antigen gene; deficiency of internalization pathways; reduction of lysosomal proteolytic function; cell cycle arrest; overexpression of drug efflux transporters; dysregulation of apoptotic pathways; activation of alternative pathways (Figure 4).

### 4.1 Target antigen downregulation, deletion, or antigen gene mutation

In the context of therapeutic pressure selection, ADCs face various obstacles that can hinder their effectiveness. These obstacles include continued repeated exposure, blocked binding to the target receptor, downregulation of the target antigen, loss of antigen expression, or antigen gene mutation.^377^ Tumor cells can develop tolerance to ADCs through repeated exposure, as seen in the case of the anti‐HER2 drug T‐DM1 and its resistance in HER2‐positive breast cancer cell lines.^378^ To study this phenomenon, researchers commonly employ experimental methods such as constructing ADC resistance models.^17^, ^379^ Interestingly, high antigen expression can paradoxically reduce the effectiveness of ADCs. This is particularly evident with the use of GO (Mylotarg®), where a high CD33 antigen load in peripheral blood is considered an independent adverse prognostic factor. When gemtuzumab is administered intravenously, CD33 saturation in peripheral blood reaches maximum levels, but saturation in the bone marrow is significantly reduced (40–90% compared with over 90% in peripheral blood samples). It appears that a high CD33 antigen load depletes gemtuzumab and limits its penetration into the bone marrow, which may explain why GO only induces remission in about 30% of patients with relapsed AML.^380^ Thus, GO should be administered at higher or repeated doses.

Truncation of the extracellular domain of the antigen or masking of extracellular matrix components may contribute to the mechanism of resistance to HER2‐ADCs. In certain tumors that overexpress HER2, there is also expression of p95HER2, which is a receptor with kinase activity that is truncated at the amino‐terminus.^381^, ^382^ An evaluation of p95HER2 expression using immunofluorescence in samples from 46 patients with HER2‐overexpressing advanced breast tumors treated with trastuzumab revealed a direct correlation between p95HER2 and trastuzumab resistance.^383^ The extracellular domain shedding of p95HER2 protein may be generated by metalloprotease.^384^ A combined therapy with a metalloprotease inhibitor and trastuzumab may overcome this mechanism. Furthermore, the expression of MUC4, a membrane‐associated mucin, is associated with trastuzumab resistance as it partially masks HER2 and hinders its binding.^385^ Inhibition of MUC4 may be potential for enhancing the sensitivity to trastuzumab therapy.^386^

Decreased antigen expression levels can also contribute to the development of ADC tolerance. Previous studies have shown that ligands such as NRG‐1β can promote heterodimerization of HER2 with HER3 and HER4, thereby reducing the effectiveness of T‐DM1.^387^, ^388^ For instance, when patients undergo front‐line trastuzumab and pertuzumab treatment, the expression level of HER2 may decrease, leading to a reduction in the therapeutic impact of T‐DM1 as a second‐line therapy.^389^ Therefore, it becomes crucial to reevaluate the HER2 status in HER2‐positive breast cancer patients to ensure optimal treatment efficacy.

### 4.2 Deficiency of internalization pathways

The entry of ADC–antigen complex into cells is a crucial step for ADCs to exert their therapeutic effects, which is accomplished through endocytosis. Endocytosis can occur through various pathways, including clathrin‐mediated endocytosis (CME), caveolin‐dependent endocytosis (CDE), and clathrin–caveolin‐independent endocytosis.^390^, ^391^ If the endocytic pathway is blocked, ADCs cannot be internalized and transported. CME involves the internalization of molecules in cells through the progressive and sequential assembly of clathrin‐coated vesicles containing various transmembrane receptors and ligands. These vesicles deform the membrane, forming a vesicular bud that matures and eventually pinches off. The resulting intracellular vesicle then undergoes clathrin uncoating and fuses with the endosome to release its contents.^392^ While CDE has been extensively researched, it is now recognized that numerous clathrin‐independent endocytic pathways exist in eukaryotic cells. Caveolae, small cave‐like invaginations in the plasma membrane, can be internalized into the cell similar to clathrin‐coated pits. These structures are sub‐domains of glycolipid rafts rich in cholesterol, sphingolipids, and caveolins, making CDE sensitive to cholesterol depletion agents like filipin and methyl‐β‐cyclodextrin. Caveolin‐1 (CAV1), essential for caveolae formation, is clustered within these membrane invaginations. Oligomerization of caveolins, facilitated by specific domains, leads to the creation of caveolin‐rich rafts in the plasma membrane. Elevated cholesterol levels, combined with the presence of caveolin scaffolding domains and the activity of dynamin2 (also involved in CME), promote the assembly and expansion of caveolar endocytic vesicles.^393^ However, given that dynamin2 is necessary for both CME and CDE, it remains uncertain whether clathrin‐coated vesicles play a role in these processes.

In preclinical models, it was observed that cell lines with acquired resistance to T‐DM1 overexpressed CAV1. N87‐TM cells (T‐DM1‐resistant N87 cells) have a high amount of intracellular caveolin and internalize trastuzumab‐ADC through CAV1 ‐ dependent endocytosis.^394^, ^395^ Over time, the colocalization of trastuzumab and CAV1 increased in N87‐TM cells.^396^ Since T‐DM1 is metabolically degraded in lysosomes, the colocalization of trastuzumab and lysosomes is reduced in these cells, leading to a decrease in the delivery efficiency of T‐DM1 to lysosomes. CAV1‐mediated endocytosis of T‐DM1 is associated with a reduced response to drugs, suggesting that CAV1 may play a role in the endocytosis of T‐DM1 and that this effect could be used to predict drug responsiveness.^396^ In the cells where ADC was enriched in CAV1 vesicles, there was a significant reduction in ADC colocalization with lysosomes. This suggests that the delivery of T‐DM1 to lysosomes is less efficient. In cell lines that are sensitive to antimelanotransferrin ADC (L49–valine–citrulline–MMAF), ADC colocalizes with lysosomes. However, in resistant cell lines, ADC colocalizes with CAV1.^71^ Although caveolae‐mediated endocytosis could be served as a potential predictive biomarker for response to T‐DM1, the strategy aiming to overcome this mechanism awaits further investigation.

### 4.3 Reduction of lysosomal proteolytic function

Lysosomes play a vital role in enabling the release of cytotoxic drugs from ADCs inside cells. Impairment in the hydrolysis or acidification function of lysosomal proteins hinders the breakdown of the linker molecule within the cell, thus preventing the release of cytotoxic drugs within the tumor cells.^397^, ^398^ In cells that were continuously exposed to T‐DM1 and developed drug resistance, there was an observed accumulation of lysosomes.^399^ However, the therapeutic effectiveness of ADCs is limited due to the reduced activity of lysosomal proteolysis, despite the presence of the drug in lysosomes.

Resistance to ADCs can also occur due to difficulties in transporting cytotoxic agents from the lysosomal lumen to the cytoplasm. When ADCs are broken down, the attached cytotoxic agents are released. However, the lysosomal membrane prevents these breakdown products from passing through, necessitating transport mechanisms to move them into the cytoplasm.^256^ To identify potential lysosomal transporters, Kinneer and colleagues conducted a phenotypic shRNA screen using an anti‐CD70–maytansine‐based ADC. This screen identified the lysosomal membrane protein SLC46A3 as a transporter that hinders the release of cytotoxic payloads from lysosomes to the cytoplasm.^400^, ^401^ Loss of SLC46A3 expression results in resistance to PBDs.^401^ Conversely, the transport efficiency of noncleavable ADCs carrying structurally distinct cys‐linker‐MMAF was unaffected by SLC46A3 attenuation.^402^

Recent studies have shown that resistance to T‐DM1 can be attributed to an increase in lysosomal pH and a decrease in lysosomal proteolytic activity.^403^ Restoring lysosomal functionality may overcome resistance to ADCs and improve their therapeutic effectiveness. Another mechanism of T‐DM1 resistance is reduced lysosomal acidification, where abnormal activity of V‐ATPase in lysosomes of resistant cells leads to a highly acidic pH that promotes optimal activity of various hydrolases and vesicular transport. This results in defects in T‐DM1 metabolism and failure to inhibit microtubule polymerization, leading to T‐DM1 resistance. However, it has been observed that another HER2‐targeted ADC containing a cleavable linker H‐MMAE can overcome this resistance.^404^ Therefore, ADCs with cleavable linkers may be able to overcome resistance caused by decreased lysosome V‐ATPase activity.

### 4.4 Cell cycle arrest

Cyclin B1 is a crucial protein that plays a vital role in activating cyclin‐dependent kinase 1 and initiating the M phase.^405^, ^406^ In T‐DM1 sensitive breast cancer cells, the expression of cyclin B is significantly higher compared with drug‐resistant cells.

Treatment with T‐DM1 results in an increase in cyclin B1 levels, leading to the onset of the mitotic catastrophe phenotype. This phenomenon, referred to as “mitotic catastrophe,” is viewed as a precursor to apoptosis, necrosis, or senescence.^407^, ^408^ However, cells resistant to T‐DM1 do not display this phenomenon unless there is a build‐up of cyclin B1. Additionally, activation of polo‐like kinase 1 hinders the mitotic process in the absence of cyclin B1.^409^ The absence of cyclin B1 causing cell cycle arrest in the G2/M phase is now recognized as a contributing factor to resistance against ADC‐induced cell death.^410^ Therefore, assessing cyclin B1 induction could serve as a pharmacodynamic predictor in ADC treatment.

### 4.5 Overexpression of drug efflux transporters

Chemoresistance in cancer cells often occurs when drugs are removed from the cytoplasm through ATP‐binding (ABC) transporters, also known as drug efflux pumps.^411^, ^412^, ^413^ These transporters can also contribute to drug resistance in ADCs because many cytotoxic drugs are substrates of these transporters. P glycoprotein (P‐gp) is identified as the primary factor contributing to resistance against vc‐MMAE‐based conjugates, irrespective of P‐gp expression levels. In cases where a drug is internalized into the cell but swiftly removed by P‐gp, the efficacy of the ADC in eradicating cancer cells is compromised.^414^, ^415^ Consequently, this mechanism leads to resistance to antimicrotubule drugs in ADCs. Studies have demonstrated that altered expression of ABCB1/MDR1/P‐gp, ABCC1/MRP1, ABCC2, and ABCG2/BCRP/MXR/ABCP can result in resistance to T‐DM1, a specific antimicrotubule drug used in preclinical models.^416^ Inhibiting the activity of these transporters in these studies restored sensitivity to T‐DM1. Of note, there is increasing evidence that conjugates such ADCs which enter the cell through endocytosis, and thereby bypass ABC transporters.

In the field of drug efflux pumps, one effective approach is to replace a drug form that can be actively pumped with a form that cannot. This can be achieved by either substituting it with a new ADC drug or modifying the payload through the use of a different load and coupling technology. For example, in the case of DM1, it was replaced with Lys–PEG4Mal–DM1, which utilized a maleimide‐based hydrophilic linker called PEG4Mal to prevent drug extrusion.^417^ Another strategy involves selecting drugs that can reduce the activity of drug efflux pumps, thereby minimizing the efflux of active metabolites.^418^

### 4.6 Dysregulation of apoptotic pathways

The modulation of apoptosis can have an impact on the sensitivity of tumor cells to ADC. In particular, resistance to GO has been linked to the overexpression of antiapoptotic proteins such as BCL‐2 and BCL‐X.^419^ Several studies have shown that PK11195 can enhance the cytotoxicity of GO in AML cells and also increase the expression of antiapoptotic proteins and drug transporters.^420^ In mouse models, PK11195 has been proven to safely enhance the effectiveness of GO against leukemia.^420^ Furthermore, Oblimersen, which targets Bcl‐2 mRNA, has been found to enhance the apoptotic activity of various antileukemia drugs, including GO, in preclinical studies.^421^ Dornan and colleagues have discovered a correlation between the expression levels of BCL‐XL and reduced sensitivity to anti‐CD79b–valine–citrulline–MMAE in non‐Hodgkin's lymphoma cell lines. In vivo data further supported the finding that the BCL‐2 family inhibitor ABT‐263 enhanced the activity of ADC.^371^

### 4.7 Activation of alternative pathways

Activation of the alternative pathway plays a significant role in contributing to ADC resistance. For instance, studies have observed that the activation of the PI3K/AKT signaling pathway leads to the development of ADC resistance. In one particular study, MK‐2206, a small molecule allosteric inhibitor of AKT, was found to enhance the sensitivity of drug‐resistant cells to GO or free calicheamicin.^422^ Other research has indicated that the PI3K/AKT pathway is activated through HER2/HER3 dimerization.^388^ Additionally, the addition of the HER3 ligand NRG has been found to counteract the effects of T‐DM1 in certain HER2‐positive cell lines.^423^

In models of acquired resistance to T‐DM1 and primary resistance to trastuzumab, a decrease in HER2 levels is observed alongside an increase in EGFR levels, which also contributes to resistance.^424^, ^425^, ^426^ However, it is important to note that solely silencing EGFR is not sufficient to reverse the drug‐resistant phenotype. Additionally, the elevated levels of EGFR were found to result in increased levels of integrins (α5β1 and αVβ3), thereby enhancing the motility and invasiveness of resistant cells.^424^

### 4.8 Judging types of causes that show resistance or tolerance to ADCs

Various methods have been utilized to identify the causes of resistance or tolerance to ADCs, depending on the specific resistance mechanism. These methods include immunohistochemical detection of antigen expression, whole‐genome sequencing to pinpoint target mutations and amplifications, assessment of proliferation‐related molecules like cyclin B1 expression levels or overexpression of antiapoptotic proteins such as BCL‐2 and BCL‐X, upregulation of ABC transporters, immunofluorescence to assess lysosome condition, proteomic analysis of proteins involved in drug efflux transport and bypass pathway activation, and evaluation of pH values within tumor cells.^394^, ^427^, ^428^ For instance, resistance to T‐DM1 can result from loss or decreased expression of HER2.^394^ ADCs that rely on consistent HER2 expression levels may face resistance if there are changes in HER2 levels. Moreover, HER2+ tumors tend to exhibit lower HER2 expression post‐treatment, with more heterogeneous expression linked to higher recurrence and lower survival rates.^429^ Therefore, it is crucial to assess the expression status of HER2 for ADCs that necessitate uniform HER2 expression. Proteomic analysis has shown that increased levels of Rab6, a protein involved in microtubule‐mediated transport, and PAK4, a protein related to cytoskeletal tension, are present in DM1‐resistant cell lines.^378^ Hence, detecting Rab6 and PAK4 levels through proteomic analysis can help identify resistance. Additionally, some ADCs feature acid‐lysable linkers that are primarily cleaved in acidic tumor microenvironments or within acidic cellular compartments like endosomes and lysosomes, while remaining stable in the bloodstream.^430^ In low pH lysosomal environments, these linkers are rapidly broken down, releasing small molecular toxins that induce cell death.^430^ Changes in pH levels can impact the efficacy of ADCs, as seen in T‐DM1‐resistant cells lacking proteolytic activity, leading to drug accumulation akin to lysosomal storage diseases. Therefore, pH‐dependent ADCs targeting EGFR, HER2, AXL, and c‐MET can be used to assess resistance by monitoring cellular pH value.^431^ By discerning the causes of resistance or tolerance to ADCs, researchers can formulate effective strategies to overcome these challenges.

### 4.9 Future design of ADCs to overcome resistance

In order to overcome ADC resistance, researchers are exploring and implementing various new strategies for ADC design. One approach is to improve the intracellular trafficking of ADCs using a bispecific antibody (bsAb) approach, such as HER2×HER3 bsAb and SORT1×HER2 bispecific ADC.^432^, ^433^ Moreover, there have been advancements in improving conventional conjugations by exploring small antibody molecules like bispecific Fab, immunosuppressive antibodies, antibody–antibiotic conjugates, and short peptide tags.^434^, ^435^ Additionally, researchers have been investigating updates to drug release patterns. Some ADCs can achieve targeted release in the hypoxic and acidic tumor microenvironment, while others can directly function in the tumor microenvironment without the need for endocytosis.^436^, ^437^ Furthermore, future ADCs may incorporate new drug‐loading ingredients or cytotoxins such as immunomodulators, nuclides, nucleic acids, and so on. These emerging technologies and theories are expected to significantly impact the development of new ADCs, enabling them to overcome drug resistance and improve efficacy and safety.

## 5 CONCLUSION AND PROSPECTS

Since the first application of ADC in 2000, ongoing research has shown that ADCs offer a new and promising treatment option for various types of cancer.^438^ When compared with traditional chemoradiotherapy, ADC drugs demonstrate superior antitumor effects due to their high specificity, effectiveness, long half‐life, minimal adverse reactions, and positive prognostic outcomes. As a result, ADCs have emerged as a prominent area of focus in global drug research and development.

While the emergence of ADCs has expanded the range of treatment options available to cancer patients, it is crucial to acknowledge that most tumors will eventually develop resistance to these drugs. Given the intricate nature of ADCs, potential mechanisms of resistance may encompass reduced antigen expression, diminished ADC transport and processing, resistance to cytotoxic payloads, and heightened drug efflux. Ongoing studies on optimizing sequential and combination therapy with ADCs will provide further insights into their optimal use. Combination drugs can effectively counter resistance and enhance treatment effects, similar to many combination therapies used in traditional chemotherapy. ADCs can also be used in combination with tyrosine kinase inhibitors, PD‐1/L1 antibody, and others.

With advancements in small molecule screening and protein recombinant molecular biotechnology, novel antibody coupling modes have emerged in drug development. These include antibody fragment–cytotoxic drug conjugate, antibody–oligonucleotide conjugate (AOC),^439^ and antibody–immunostimulatory conjugate.^440^, ^441^ Notably, the first AOC incorporating oligonucleotide‐based drugs has progressed to clinical trials.^442^ While traditional ADCs rely on internalization into tumor cells for cytotoxin release, limiting research to targets with highly expressed internalized antigens, recent focus has shifted to noninternalizing antibody‐coupled drugs with extracellular payload release mechanisms. This approach broadens the spectrum of cancer targets by eliminating the need for high antigen expression and inefficient internalization.^443^ Moving forward, enhancing clinical efficacy and tolerability remains a priority. Understanding the interplay between antibody coupling drug structures, clinical activity, and mechanism of action is essential for optimizing design elements, such as exploring antibody versatility and developing more stable linkers. The discovery of new antigens and cytotoxic drugs will be critical in enhancing drug efficacy.

## AUTHOR CONTRIBUTIONS

Jun He and Xianghua Zeng collected the literatures and drafted the manuscript. Chunmei Wang drew the figures and tables. Corresponding authors, including Yongsheng Li and Enwen Wang, provided their corrective comments and tips. Yongsheng Li revised the manuscript. All authors collaborated to write the article and approved its publication.

## CONFLICT OF INTEREST STATEMENT

The authors have declared that no conflict of interest exists.

## ETHICS STATEMENT

Not applicable.

## Data Availability

Not applicable.
